# Mesophotic fish communities of the ancient coastline in Western Australia

**DOI:** 10.1371/journal.pone.0250427

**Published:** 2021-04-21

**Authors:** Leanne M. Currey-Randall, Ronen Galaiduk, Marcus Stowar, Brigit I. Vaughan, Karen J. Miller

**Affiliations:** 1 Australian Institute of Marine Science, Townsville, Queensland, Australia; 2 Indian Ocean Marine Research Centre, Australian Institute of Marine Science, University of Western Australia, Crawley, Western Australia, Australia; California Academy of Sciences, UNITED STATES

## Abstract

Marine diversity across the Australian continental shelf is shaped by characteristic benthic habitats which are determined by geomorphic features such as paleoshorelines. In north-western Australia there has been little attention on the fish communities that inhabit an ancient coastline at ~125 m depth (the designated AC125), which is specified as a key ecological feature (KEF) of the region and is thought to comprise hard substrate and support enhanced diversity. We investigated drivers of fish species richness and assemblage composition spanning six degrees of latitude along sections of the ancient coastline, categorised as ‘on’ and ‘off’ the AC125 based on depth, across a range of habitats and seafloor complexity (~60–180 m depth). While some surveyed sections of the AC125 had hard bottom substrate and supported enhanced fish diversity, including over half of the total species observed, species richness and abundance overall were not greater on the AC125 than immediately adjacent to the AC125. Instead, depth, seafloor complexity and habitat type explained patterns in richness and abundance, and structured fish assemblages at both local and broad spatial scales. Fewer fishes were associated with deep sites characterized by negligible complexity and soft-bottom habitats, in contrast to shallower depths that featured benthic biota and pockets of complex substrate. Drivers of abundance of common species were species-specific and primarily related to sampling Areas, depth and substrate. Fishes of the ancient coastline and adjacent habitats are representative of mesophotic fish communities of the region, included species important to fisheries and conservation, and several species were observed deeper than their currently known distribution. This study provides the first assessment of fish biodiversity associated with an ancient coastline feature, improving our understanding of the function it plays in regional spatial patterns in abundance of mesophotic fishes. Management decisions that incorporate the broader variety of depths and habitats surrounding the designated AC125 could enhance the ecological role of this KEF, contributing to effective conservation of fish biodiversity on Australia’s north west shelf.

## Introduction

Studies on mesophotic and deep-water fish communities in tropical waters are rare because of limitations associated with surveying remote, deep marine environments [1, 2]. As a result, the distribution, abundance and habitat associations of fish species that occupy these ecosystems are less known compared to shallow water ecosystems [2–5]. Fish diversity generally declines with increasing depth from shallow to deep marine environments [6, 7], which is often related to the decline of important, light-dependent habitats (e.g. coral reefs, macroalgal dominated benthos) as depth increases [8]. However, the increasing use of remote survey technologies has enabled more detailed study of lesser-known mesophotic (60–180 m depth) and aphotic habitats beyond the limits of traditional diver-based techniques. Some of these surveys have revealed areas of unique and rich biodiversity in deep water [9, 10].

Mesophotic and deep-water fish communities are of conservation interest, since species assemblages may be distinct from those in shallower depths [11], be characterised by a high degree of endemism (e.g. [12]) or be long-lived [13]. Therefore, they may be particularly sensitive to environmental pressures such as global warming, ocean acidification and fishing [14–16]. However, there is also some suggestion that deeper habitats may serve as refuges for more mobile species from warming sea temperatures [17, 18]. To ensure effective protection and environmental impact mitigation strategies for mesophotic and deep-water fish communities it is critical to understand the patterns and drivers of distributions in these communities, their associations with shallower systems and ultimately, their ecological processes.

Like deep marine habitats in the rest of the world, the offshore waters and associated biodiversity of the deeper marine habitats of the North West Shelf (NWS) of Australia are poorly studied. The region is characterised by an expansive continental shelf that extends from the continental margin; 40% of the north-west Australian exclusive economic zone is at depths of less than 200 m [19]. The NWS contains 13 key ecological features (KEFs) recognised for unique geomorphology, unique marine life, high biological productivity such as upwelling, or species or habitats with regionally important ecological roles or high biodiversity/endemism [20, 21]. However, detailed information on habitats and fish diversity for most of the KEFs is scarce, especially for KEFs in deeper water or remote locations. Australia’s resource-rich NWS and its KEFs face pressures from industrial activities (e.g. oil and gas extraction), regional development, commercial and recreational fishing activities and climate change. In this context, it is important to document the region’s biodiversity, particularly the abundance, distribution and assemblage structure of fish fauna to best manage these pressures.

**Fig 1:**
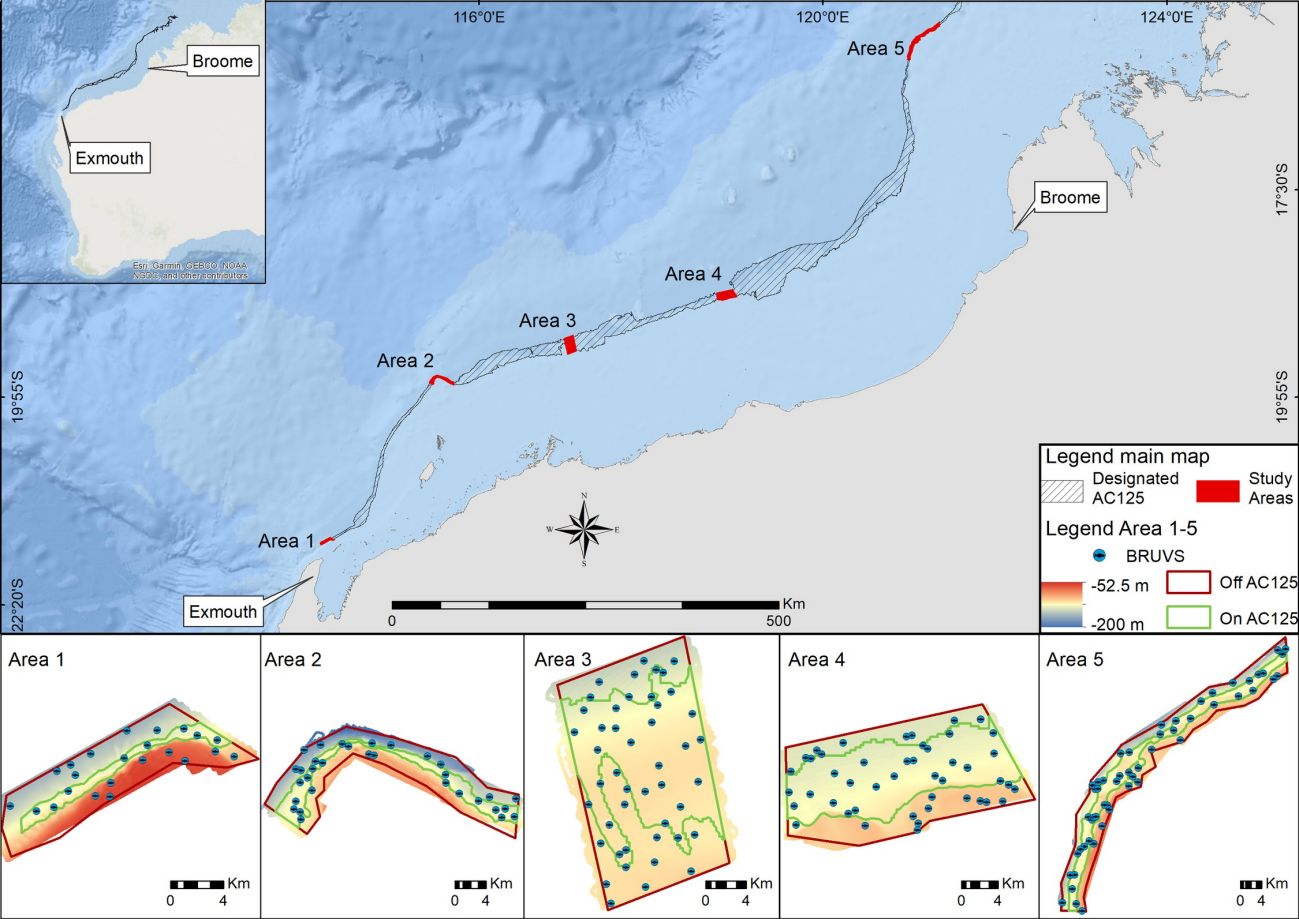
Location of sampling within the five sampling areas along the designated AC125 (crosshatched polygon), depth and BRUVS deployments (points). Within the designated AC125, on AC125 is defined as between 115-135m depth based on existing bathymetry for this region [42]. This map was generated using ESRI software.

Along the NWS, the main bathymetric feature, formed by erosional processes, is a terrace with its base at 125 m depth and an escarpment rising up to ~90 m [22, Fig 1], though due to lack of fine scale bathymetry and detailed studies for the region the present day underwater topography and geomorphic history of this feature is unknown for much of its extent. This submerged ancient coastline at the 125 m depth contour (herein referred to as the ‘designated AC125’) off the north-west Australian coast has been designated as a KEF because it is a unique seafloor feature comprising areas of hard substrate that represent distinct benthic habitats for associated fish assemblages [20]. Its topographic variation is thought to play a role in altering local oceanographic processes, resulting in nutrient upwelling and associated increased regional productivity that may support a high biodiversity of fish, birds and marine mammals [20]. Formation of the designated AC125 coincided with the worldwide eustatic sea-level stillstand at about 130 m below present sea level which preceded the Holocene transgression about 17,000 years ago [23]. As a result, the designated AC125 is the most prominent of terraces and steps from the Holocene on the NWS as well as the largest KEF in the NWS, spanning over 1,500 km [20]. Due to its remoteness and depth, it is also one of the least studied KEFs within this region [24]. To date, no systematic study has documented the different habitats and their fish communities along its extent, despite the recognised importance of palaeoshorelines in shaping biodiversity [25–27]. Likewise, these fish communities have not been compared in a regional context to the fish and benthic communities of adjacent areas.

Early scientific work on fish communities of the NWS encompassing the designated AC125 documented species captured by commercial and research trawling up to the late 1970s [28]. In the late 1970s Australia’s Commonwealth Scientific and Industrial Research Organisation (CSIRO) undertook dedicated research cruises to document the fish fauna of the northwest, although the outputs of these surveys were mainly taxonomic in nature rather than reports on fish–habitat associations (e.g. [29]). CSIRO’s 1982 and 1997 data were integrated into more recent trawl, tow video and sled sampling to document fishes of the Dampier and Montebello Australian Marine Parks [30]. Other studies of fish assemblages of the NWS have focused on areas of specific biological interest, for example the Rowley Shoals [31–33], Ningaloo Reef World Heritage Area [34, 35], Browse Island [20, 36], Sahul Banks [37] and Rankin Bank [38] The broader NWS also supports the most valuable commercial finfish fisheries in Western Australia, the Pilbara trap, line and trawl fisheries and the Northern Demersal Scalefish Fishery, which are worth more than $10 million annually with a catch of 3000–4000 tonnes per year [39]. These fisheries have also been a focal point of research documenting the catch and biology of fish fauna of the NWS, in particular the high market value snappers, emperors and cods on which the fishery is based [15, 40, 41]. While some inferences can be drawn about fish communities of the designated AC125 based on sampling undertaken in relevant depths in these targeted regional studies, broader habitats and their associated fish communities throughout the region have not been documented.

This study aimed to improve the current knowledge of mesophotic (60–180 m depth) fish biodiversity and distribution patterns along the designated AC125 to identify the significance of these areas regionally for management and conservation. The designated AC125 sections sampled were further categorised into positions ‘on’ the AC125 (125 m contour ± 10 m) and adjacent habitat ‘off’ the AC125 (shallower or deeper). Specifically, we aimed to answer: 1) Does species composition and richness of fishes on the AC125 differ from the habitats immediately adjacent? 2) Is there latitudinal variation in fish communities along the AC125? 3) How does species composition and richness compare with other habitats regionally? and 4) Do species of fishery and conservation importance occur? We document the species richness and composition of fish assemblages from five representative areas along the designated AC125 and a range of habitats and seafloor complexity. We filled in knowledge gaps by predicting species richness for the region to elucidate the role of the AC125 in regional dynamics of fish biodiversity on the NWS of Australia. Our findings will improve future biodiversity-focused environmental management efforts for the NWS by helping policymakers and managers to identify conservation strategies and to inform decision-making processes.

## Materials and methods

### Surveys and sampling locations

The designated AC125 extends for more than 1500 km along the coast of Western Australia (Fig 1) and we selected five representative ‘Areas’ spaced along approximately 1000 km of this region (as a proxy for latitude). Within each Area on the designated AC125, we further derived the 125 m contour ± 10 m depth on each side as ‘on AC125’, with remaining areas defined as ‘off AC125’, to compare fish diversity and abundance on the AC125 as well as in adjacent habitats off the AC125 contour (Fig 1). The selection of study Areas was based on existing coarse-scale bathymetry for this region [42] and relative to other known fish data sets from the NWS to facilitate regional comparions. Based on existing bathymetry, Areas 1, 2 and 5 were expected to have a high proportion of hard substrate and therefore have relatively higher diversity and enhanced fish species richness. In contrast, Areas 3 and 4 were expected to be more representative of the broad region, which is characterised primarily by soft-sediment seafloor and low structural complexity. The on/off AC125 polygons were used as a priori features for the sampling design within each study Area. We used a spatially balanced, unequal inclusion probabilities survey design with generalized random tessellation (GRTS; [43]) to allocate baited remote underwater video station (BRUVS; [44–46]) deployment sites within each Area. This type of survey planning allowed for stratification of the sampling effort across the AC125 features and five study Areas, while ensuring that the data were sufficient to build spatial predictive models over as large an extent of the region as possible while maintaining robust results. We surveyed fish communities between March and October of 2019, with BRUVS deployed between 62.1 m and 181.4 m depth during daylight hours. Our surveys along the length of the designated AC125 included research in the Kimberly Marine Park which was conducted under an Australian Marine Park Activity Permit (PA2019-00019-1) authorised by the Australian Government Director of National Parks.

BRUVS comprised GoPro Hero4 cameras (30 fps 1920 × 1080 pixel resolution, medium field of view), which were baited with 1 kg of crushed pilchards (*Sardinops sagax*), and deployed for 60 minutes with a minimum distance of 500 m between deployments to avoid potential overlap of bait plumes and movement of fish between BRUVS [47]. BRUVS were fitted with artificial white lights (natural day white light, ~ 5600 K) which was optimal for identifying diurnal mesophotic fishes at depth for this study [48]. There were 204, one-hour BRUVS deployments across the five Areas.

We analysed videos to determine species richness and relative abundance (as MaxN: [49–52]). MaxN is the maximum number of individuals of each species observed within a single frame during a 60-minute deployment on the seabed. Unidentified taxa (identified only to genus) and fishes that were difficult to identify on video at depth or subject to variability in identification by readers, were grouped for statistical analysis (S1 Table).

We derived seabed composition and data on benthic biota from the BRUVS field of view to determine the proportion of biotic and abiotic cover (Table 1) on each deployment.

**Table 1:** Spatial and habitat predictors and responses used in analyses of marine communities along the designated AC125.

**Predictor **	**Description**
Area (a proxy for latitude)	Area1, Area2, Area3, Area4, Area5
AC125 position	On (sites within a priori polygon at 115-135m depth based on existing bathymetry [42]); Off Shallow (sites <125 m deep outside ‘On AC125’ polygon); Off Deep (sites >125 m deep outside ‘On AC125’ polygon)
Depth	metres
Biotic cover: Benthic biota (Benthos)	0–100% cover including bryozoans/encrusting animals, gorgonians, sponges, soft corals, hydroids and whips
Abiotic cover: Boulder/calcareous reef %	None, low, high
Abiotic cover: Rubble %	0–100%
Abiotic cover: Gravel %	0–100%
Abiotic cover: Mud/silt %	None, low, high
**Response**	**Description**
Species richness	Number of fish species/groups
Relative abundance	Summed MaxN, number of individual fish
Relative abundance of common species (five most abundant species)	Summed MaxN of *Pristipomoides multidens*, *Carangoides caeruleopinnatus*, *Nemipterus bathybius*, *Lagocephalus lunaris*, *Epinephelus areolatus*

### Drivers of fish species richness, overall abundance, common species abundance and community structure

We used generalized additive mixed models (GAMM) with the *gamm4* package in R [53] using a log (richness and abundance) Tweedie distribution to test whether fish species richness, relative abundance and abundance of common species were higher on the AC125, or whether a latitudinal gradient (by Area) was evident. Position relative to the AC125, Area, depth, and habitat predictors (Table 1), were included in a full-subsets information theoretic model selection process based on the Akaike information criterion (AIC) corrected for small-sample bias [54, 55]. We fitted all possible combinations of models including up to three predictors and excluded any models where predictors were correlated by more than 0.28 [55] Best-supported models were those with ΔAIC_c_ values <2 and with the fewest terms [54]. Response and predictors were transformed where necessary before analysis, including pooling some abiotic substrate categories (none, low and high) due to fewer data and non-normal distributions (i.e. boulder/calcareous reef and mud/silt). Visibility was incorporated as a null term (smoothed predictor) in all models to remove any effect of video readability. Highly correlated predictors (e.g. sand substrate and bare benthos correlated with all other substrate/benthos categories (Table 1), and were observed at all Areas) were removed from analyses.

To test the importance of predictors as drivers of fish community structure, species present on at least 10 BRUVS (53 species) were included in distance-based redundancy analyses (dbRDA) for each Area. The distance matrix (dissimilarities) was calculated on fourth-root transformed MaxN abundance data using the site-standardised (Manhattan distance) extended dissimilarity (xdiss() in library mvpart; [56]). The eigenvalues obtained in the principal components analysis were used with the capscale function to perform the ordination via the *vegan* package [57]. Capscale uses non-Euclidean dissimilarity indices yet remains strictly linear and metric. We measured the variation accounted for by each axis by permutation tests which assessed significance of each spatial and environmental predictor using pseudo-F values. The envfit() function (library vegan) identified the direction of abundance vectors for fish species (in the k-dimensional ordination space) that had maximal correlation with spatial and environmental covariates. We standardised the species abundance (raw scale) by survey matrix relative to other entries using the Hellinger method [58]. Site scores and weighted averages from ordinations of the multidimensional response into two dimensions were produced in the *vegan* package ([57]; rda function). The model enabled partitioning of the multivariate species variation explained by each predictor, and the unconstrained, unexplained variation. We used envfit in *vegan* to find the direction of species abundance vectors (in the k-dimensional ordination space) that had maximal correlation with predicting covariates. A threshold correlation of *p* < 0.001 was set to select significant species vectors for biplots. The dissimilarity (distance) matrix comprised 53 fish species at 203 BRUVS (one of the 204 BRUVS recorded no fish) sites (xdiss) and was used as the response in the models:

xdiss∼AC125+Area+depth+benthos+rubble+mud/silt+boulder/reef+visibility (1)

xdiss∼AC125+condition(Area+depth+benthos+rubble+mud/silt+boulder/reef+visibility) (2)

To test the difference in the fish community between sites on and off the AC125, model (2) included AC125 with the “condition ()” term used for all other predictors in the redundancy analysis.

We included month in analyses to account for any temporal variation but subsequently removed it because it was not a significant predictor. Using the AC125 spatial polygons derived from existing bathymetry, BRUVS deployments were categorised as ‘On’ or ‘Off’ the AC125, with the ‘Off’ AC125 deployments split into ‘Off Shallow’ or ‘Off Deep’ (Table 1). All analyses were conducted in R [59].

### Environmental predictors for spatial modelling

Bathymetry was mapped in each sampling area with an R2Sonic 2026 multibeam echo sounder (Fig 1). The individual bathymetry rasters were mosaiced at 10 m cell resolution and used to produce a variety of secondary (textural) datasets using terrain analysis techniques [60]. Calculations were run on a number of cells surrounding each pixel in varying size neighbourhoods to reveal textural differences, thus creating a derivative dataset (Table 2). These datasets may correlate with and act as surrogates for explaining fish diversity patterns [61–64]. We used custom-written Python code applied in ArcGIS 10.6 to derive environmental predictors of fish species richness across the five study Areas of the AC125. In addition, another set of regional environmental predictors was derived using the approach described above and the coarse-scale bathymetry raster (250 m pixel) available for Australia (see [43]).

**Table 2:** Data sets derived from multibeam bathymetry that were used as environmental surrogate variables for spatial predictive modelling of fish richness.

Benthic habitat predictor variable	Description	Predictor variable code in the model
Bathymetry	Water depth in metres, interpolated from multibeam data to 10 m resolution.	depth
Slope	First derivative of elevation. Average change in elevation, calculated on a 3×3-pixel neighbourhood (steepness of the terrain).	slp
Aspect	Azimuthal direction of the steepest slope (0–360°), calculated on a 3×3-pixel area.	asp
Overall curvature	Combined index of profile and plan curvature (see below).	curv
Profile curvature	Second derivative of depth: concavity/convexity parallel to the slope, calculated for a 3×3-pixel neighbourhood.	prof
Plan curvature	Second derivative of elevation: concavity/convexity perpendicular to the slope, calculated for a 3×3-pixel neighbourhood.	plan
Depth range across various spatial neighbourhoods	Local relief: maximum minus minimum depth within spatial neighbourhood’s equivalent in width to 5, 10, 25, 50 pixels.	rng5, rng10, rng25, rng50
Variability of depth across various spatial neighbourhoods	Standard deviation of depths within spatial neighbourhood’s equivalent in width to 5, 10, 25, 50 pixels.	std5, std10, std25, std50
Average depth across spatial neighbourhoods	Hypsometric index: average of depth within spatial neighbourhood’s equivalent in width to 5, 10, 25, 50 pixels.	hyp5, hyp10, hyp15, hyp50

Two sets of predictors were derived: one set for the AC125 spatial model using mosaiced 10 m pixel bathymetry rasters collected in this study, and one set using 250 m pixel coarse-scale bathymetry.

### Spatial modelling of fish species richness

We used the random forest (RF) ensemble machine-learning algorithm [65] to model the diversity (species richness) of fish based on BRUVS data across the five surveyed AC125 Areas using all derived predictors (Table 2). To understand the role of the AC125 within the broader north-west region, we also created a regional RF model of species richness using both our BRUVS fish richness data and the historical fish data from the Australian Institute of Marine Science’s BRUVS database (https://apps.aims.gov.au/metadata; https://eatlas.org.au; https://northwestatlas.org/nwa). RF is a commonly used algorithm for spatial modelling [66, 67]. It can fit both linear and complex non-linear models very efficiently without being prone to overfitting because it includes the results of multiple trees from bootstrap samples of the training data and reduces bias through random predictor selection [68]. The models have high accuracy compared to other comparable methods and provide ecologically interpretable outcomes [65, 69].

We used the *ModelMap* [70] package in R to fit, validate and predict the RF models. Robust species distribution modelling requires validation of the developed models with test data [66, 67]. Thus, we withheld a random sample of 30% of the fish richness data to use for model performance estimates (testing set) and used 70% of it (training set) for model development. We used the fitted RF models to predict richness from the test dataset, then compared the predicted values to the observed values from the test dataset using a Spearman correlation test and root mean square deviation (RMSD). At the end of the fitting process, we extracted the relative importance of all predictor variables, ranked using the mean decrease in accuracy metric, by randomly permuting each predictor value in the “out of bag” observations for each tree. The larger the mean decrease in accuracy of each predictor, the greater the influence it has on prediction accuracy and the greater its importance as a predictor variable in the model. We then produced partial response plots for the top four environmental predictors to examine their nature and magnitude of influence on fish species richness.

As the environmental covariates used in the modelling process cover the entire study area, they were used to predict fish richness in the five AC125 Areas (on the 10 × 10 m grid) as well as across the broader north-west region (on the 250 × 250 m grid) based on the statistical relationships we developed. Due to the relative spatial sparsity of the regional field data we mapped the coefficient of variation by dividing the standard deviation of each pixel by the mean of the pixel [70] to visualise the uncertainty associated with our model predictions for this vast region.

## Results

### Trends in fish species richness and abundance

**Fig 2:**
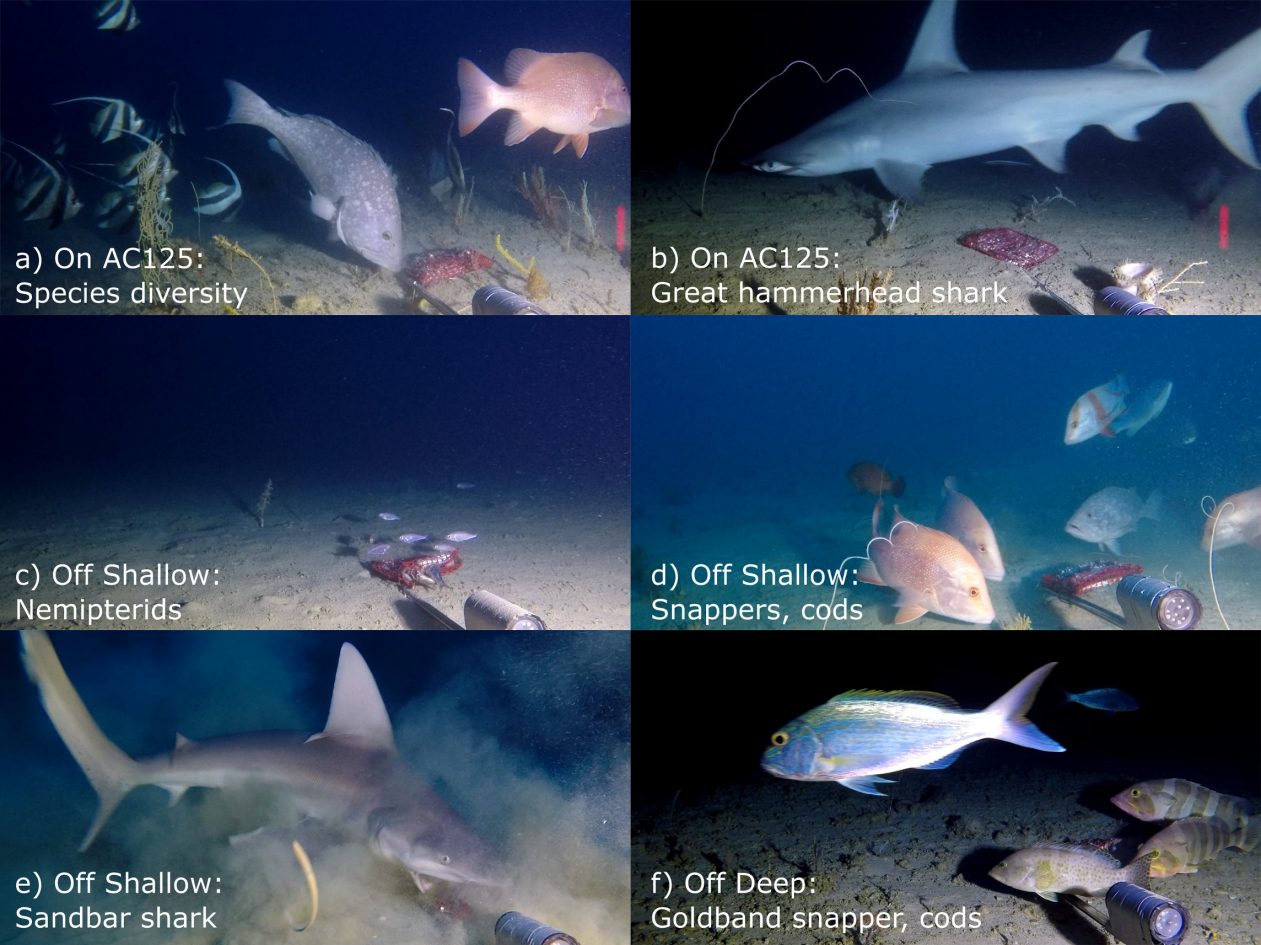
The five areas explored using BRUVS revealed a variety of fish communities. (a, b) Area 2, (c) Area 3, (d) Area 4, (e, f) Area 5.

We observed a total of 2,874 individuals, representing 39 families and 141 species/groups (S1 Table) from the 204 BRUVS deployed on and off the AC125 within the five Areas (Fig 2). Species found on the AC125 comprised 53% of all species recorded (75 of the 141 species/groups), 76% were observed off AC125 on shallow BRUVS (107 species/groups) and 45% off AC125 in deep sites. Sponge and gorgonian-dominated habitat patches supported a higher diversity of reef-associated fish species (Fig 2A and 2D), with commercially important snappers (Lutjanidae), cods (Epinephelidae) and emperors (Lethrinidae) particularly abundant in areas of outcropping consolidated substrate in Area 5 (Fig 2F). Extensive areas of fine silty substrate or soft-sediment habitats with a sparse benthic community were characterised by low diversity of fish, and often dominated by small-bodied species such as threadfin breams (*Nemipterus* spp. Fig 2C), and a number of wide-ranging shark species (e.g. great hammerhead shark *Sphyrna mokarran* and sandbar shark *Carcharhinus plumbeus*, Fig 2B and 2E). We sighted a total of 170 sharks and rays from 19 species on 60% of BRUVS deployments.

**Fig 3:**
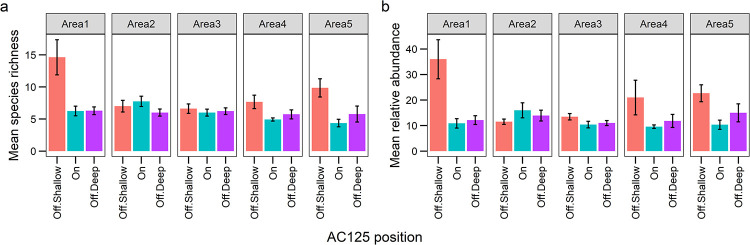
Mean (± SE) fish species richness (a) and relative abundance (MaxN, b) per BRUVS deployment observed on and off the AC125 in five sampling areas.

Area 1 had the greatest number of species and individuals (78 species, 460 fish) compared to all other Areas, and sampled across the broadest depth range (62.1–163.9 m, depth range of ~102 m, Table 3, S1 Table). Areas 1 and 5 included the shallowest depths of all areas (<100 m), and BRUVS in Areas 3 and 4 sampled across a narrow depth range of only 36 m, all consequences of characteristics in the local bathymetry (Table 3). Shallow sites off the AC125 at Area 1 supported the most species and individuals, followed by shallow sites off the AC125 in Area 5 (Fig 3), likely related to the higher relative proportion of boulder/reef substrate and cover of benthic biota (habitat complexity) in these areas (S1 Fig). Mean species richness and mean overall relative abundance was higher at shallow sites off the AC125, in all Areas except Area 2, where there was a trend for more species (and higher abundance) on the AC125 (Fig 3). Overall, sites on the AC125 had less than 13% benthos cover and <1% cover of boulder/reef substrate on average, compared to ~27% and ~15% at shallow sites off the AC125 respectively.

**Table 3:** Depth (m) of deployments in each sampling area along the designated AC125.

Area	mean	median	minimum	maximum	range
1	117.3	129.5	62.1	163.9	101.8
2	135.1	129.9	100.3	181.4	81.1
3	124.4	119.1	113.0	148.7	35.7
4	127.7	131.8	105.5	141.4	35.9
5	123.7	124.8	86.0	157.1	71.1

**Fig 4:**
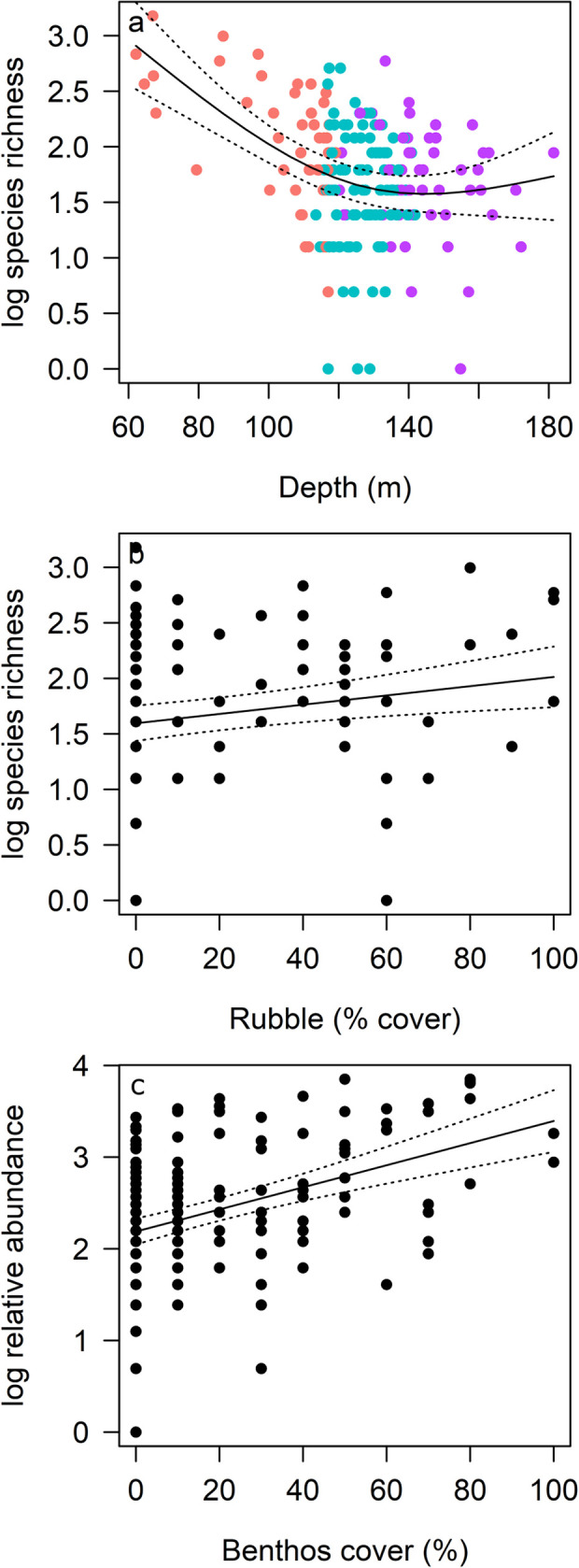
GAMM predicted trends in log transformed fish species richness (a, b) and relative abundance (c) with depth, proportion of rubble and cover of benthic biota (benthos) from best models. Lines indicate the fit of the best-approximating model with 95% confidence intervals (dotted lines). (a) is coloured by AC125 position, highlighting the relationship with depth (pink: Off AC125 shallow; blue: On AC125; purple: Off AC125 deep).

Position on the AC125, Area or their combination (additive or interaction) were of little importance in driving richness and abundance: models including these factors (R19, R21, A55, A60, A64) ranked lower than models that included other predictors (Table 4). Species richness (log transformed) was best explained by depth and rubble substrate cover (GAMM: R1, Table 4, 23% variance explained), although the addition of gravel or a model with boulder/reef and rubble substrate also explained a similar variance (models R2 and R3). Shallower depths supported greater species richness, which generally declined with increasing depth to approximately 140 m, after which more variation was observed (Fig 4A). More species were observed with increasing rubble cover (Fig 4B), thus fewer species were associated with deep sites characterised by soft sediment. These patterns also reflect higher richness in Areas 1 and 5, where habitat in shallow depths comprised higher proportions of complex substrate than other Areas (S1 Fig). Similarly, we identified three top models for relative abundance (summed MaxN), with the most parsimonious model predicting higher abundance with increased benthic biota cover (A1, 17% variance explained, Table 4, Fig 4C). Thus, depth and habitat were more important than Area or position on or off the AC125 for explaining patterns in richness and abundance.

**Table 4:** Top three GAMM model fits and fits exclusively with AC125 and area, examining the effects of predictors on log transformed richness (R) and relative abundance (A, summed MaxN).

Model name	Model	AIC_c_	ΔAIC_c_	wAIC_c_	R^2^	edf
R1	log richness ~ depth + rubble	286.155	0.000	0.311	0.232	7.01
R2	log richness ~ depth + gravel + rubble	287.434	1.279	0.164	0.235	7.99
R3	log richness ~ boulder/reef + rubble	287.576	1.421	0.153	0.220	6.31
R19	log richness ~ AC125 + Area	301.023	14.868	0	0.188	8.8
R21	log richness ~ AC125 × Area	302.964	16.809	0	0.252	16.73
A1	log abundance ~ benthic biota	390.717	1.323	0.205	0.170	3
A2	log abundance ~ boulder/reef + rubble	389.394	0.000	0.398	0.193	5
A3	log abundance ~ benthic biota + gravel	391.386	1.992	0.147	0.179	4.20
A55	log abundance ~ AC125	404.537	15.143	0	0.139	5.77
A60	log abundance ~ AC125 + Area	408.508	19.114	0	0.155	9.35
A64	log abundance ~ AC125 × Area	415.833	26.438	0	0.203	17.45

Predictors included AC125 position, Area, depth, proportional cover of benthic biota, mud/silt, gravel, rubble and boulder/reef substrate. AIC_**c**_ is the small-sample bias-corrected form of Akaike’s information criterion, ΔAIC_**c**_ is the Akaike difference, and w is the Akaike weight. Models shown are the best fitting models ΔAIC_**c**_ <2. Estimated R^**2**^ value and the total model estimated degrees of freedom (edf) are provided. Visibility was used as a null term in the analysis.

### Trends in common species abundance and community structure

**Fig 5:**
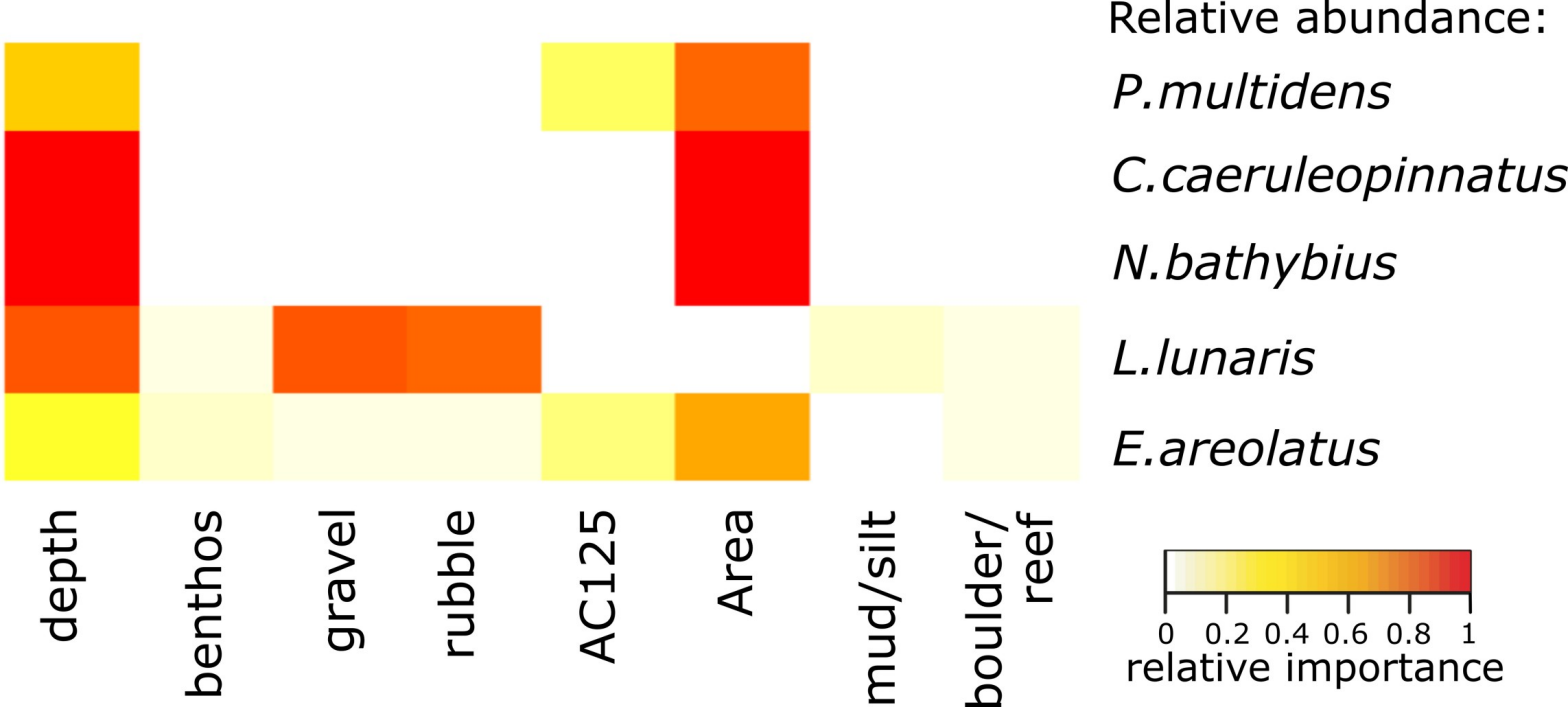
Heatmap indicating the key predictors from the GAMM models of most common species relative abundance. Colours indicate relative importance.

**Fig 6:**
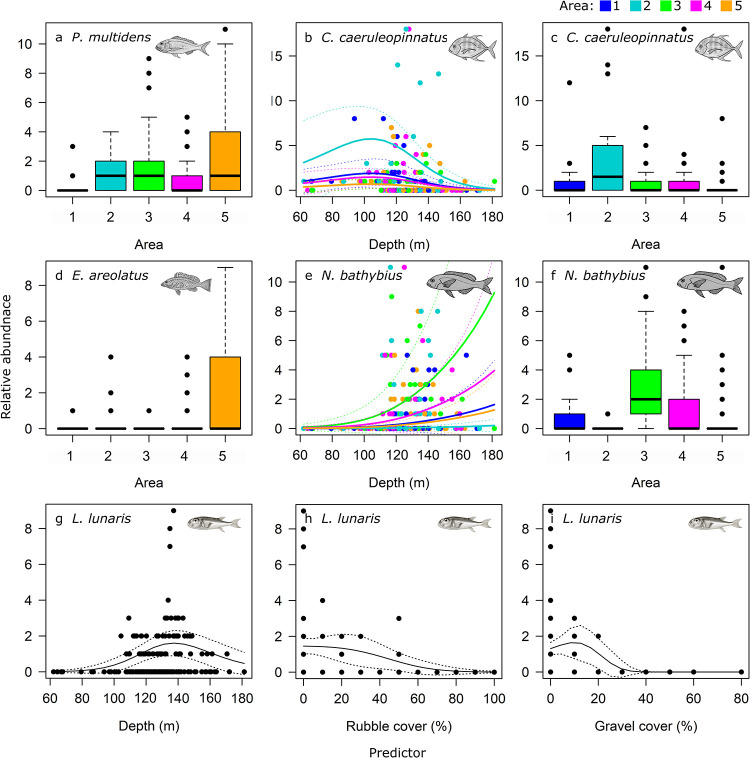
GAMM predicted trends in relative abundance (MaxN) per BRUVS deployment of common species: *Pristipomoides multidens* (a); *Carangoides caeruleopinnatus* (b, c), *Epinephelus areolatus* (d), *Nemipterus bathybius* (e, f) and *Lagocephalus lunaris* (g, h, i), by Area, depth and proportion of rubble and gravel cover from best models. Lines indicate the fit of the best-approximating model with 95% confidence intervals (dotted lines). a-f are coloured by Area 1–5.

For the five most common species by abundance, Area and depth were the strongest drivers in the models of relative abundance (Table 5, Figs 5 and 6). While richness was higher in Area 1, the commercially important *Pristipomoides multidens* (goldband snapper) and *Epinephelus areolatus* (yellowspotted rockcod) were more likely to occur in greatest abundance in Area 5 (Figs 5, 6A and 6D), while *Carangoides caeruleopinnatus* (onion trevally) showed an affinity to Area 2 and depths between 100 and 140 m (Fig 6B and 6C). At depths >120 m, models indicated *Nemipterus bathybius* (yellowbelly threadfin bream) was abundant, particularly in Area 3, and numbers of *Lagocephalus lunaris* (rough golden toadfish) was predicted greatest at ~140 m depth with <50% rubble and <30% gravel cover (Fig 6E–6I).

**Table 5:** Top GAMM models examining the effects of predictors on most common species relative abundance at the five areas.

Model name	Model	AIC_c_	ΔAIC_c_	wAIC_c_	R^2^	edf
P1	*Pristipomoides multidens* ~ Area	677.046	1.397	0.256	0.133	7.77
P2	*Pristipomoides multidens* ~ depth + Area	675.649	0	0.516	0.133	9.17
P3	*Pristipomoides multidens* ~ AC125 + Area	677.298	1.65	0.226	0.145	9.87
C1	*Carangoides caeruleopinnatus* ~ depth + Area	577.868	0	0.998	0.155	7.89
N1	*Nemipterus bathybius* ~ depth + Area	523.188	0	1	0.205	9.83
L1	*Lagocephalus lunaris* ~ depth + gravel + rubble	417.629	0	0.3	0.055	10.25
E1	*Epinephelus areolatus* ~ Area	375.293	0	0.360	0.163	6
E2	*Epinephelus areolatus* ~ depth + Area	375.495	0.202	0.326	0.179	7.74
E3	*Epinephelus areolatus* ~ AC125 + Area	376.288	0.995	0.219	0.178	8

Predictors included: AC125 position, Area, depth, proportional cover of benthic biota, mud/silt, gravel, rubble and boulder/reef substrate. All other conventions follow Table 4.

**Fig 7:**
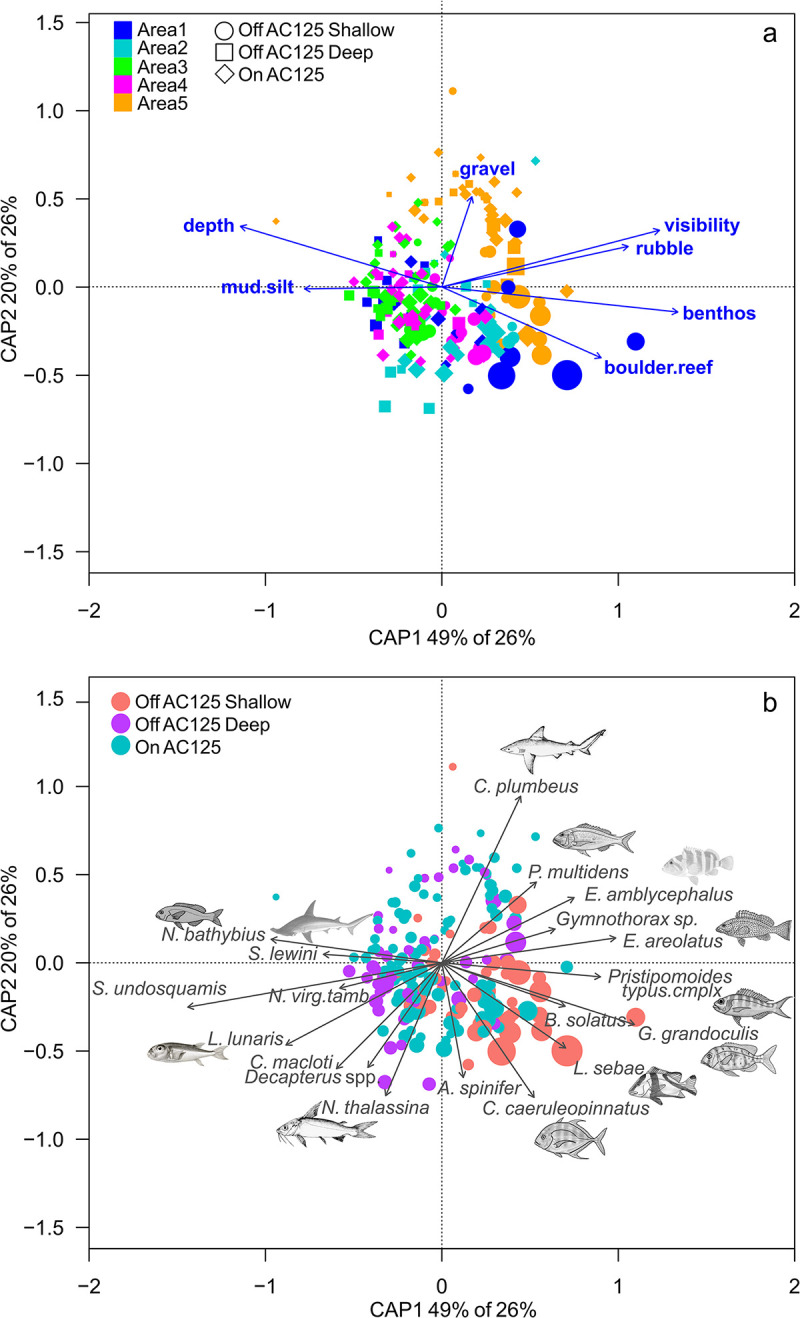
Redundancy analysis (dbRDA via capscale) biplot for fish species occurring in at least 10 BRUVS across the five areas, by AC125 position, depth, cover of benthic biota (benthos) and substrate (mud/silt, gravel, rubble, boulder/reef), and visibility. AC125 position is indicated by squares, circles and diamonds, coloured by Area (a), with the same analysis coloured by AC125 position (b). Weighted averages of site scores (points) are scaled by site richness and significant species vectors (p < 0.001) correlated with linear constraints are indicated.

AC125 position (on vs off AC125) was not a significant factor in explaining the community composition of fishes (*p* < 0.05, Table 6), with no clear separation between site scores in the ordination (spread of points, Fig 7). Sites with the highest species richness were positioned in depths shallower than the AC125. Fish community composition varied significantly with Area (latitude), depth, and cover of rubble and boulder/reef substrate (dbRDA model, explaining 26% of the distance variation, Table 6). AC125 position was not a strong driver of fish community composition, with only 1% of variation attributed to this factor (all other predictors modelled as condition term, model 2). Higher species richness (point size, Fig 7) was associated with shallow sites with greater structural habitat complexity (boulder/reef, Fig 7A). Variation between Areas was likely not related to a latitudinal gradient (south–north), rather Area 5 was distinct from the other Areas (Fig 7), characterised by sites with a high proportion of hard substrate (boulder/reef, rubble, gravel, S1 Fig), and lower turbidity (not significant). All Areas included multiple substrate types except for Area 3, which was dominated by soft sediment (sand and mud/silt) and the lowest proportion of live benthic biota (S1 Fig).

**Table 6:** Permutation tests of the effects of spatial and environmental predictors on the dissimilarity matrix of fish species.

Predictor	Df	SS	Pseudo-F	Pr(>F)
Area	4	0.12	4.57	**0.001**
Depth	1	0.02	3.27	**0.001**
AC125 position (On, Off shallow, Off deep)	2	0.02	1.37	0.101
Benthos	1	0.01	1.33	0.195
Visibility	1	0.01	0.99	0.410
Gravel	1	0.01	0.84	0.567
Mud/silt	1	0.01	1.54	0.100
Rubble	1	0.01	2.25	**0.018**
Boulder/reef	1	0.01	1.85	**0.040**
Residual	190	236.04		

Significant predictors (see Table 1) are indicated in bold and represent where the modelled relationship was stronger than the randomly permuted relationships (at alpha = 0.05, beta = 0.01), SS is the sums of squares.

Fishes associated with deep sites with low habitat complexity in Areas 1, 3 and 4 included *Nemipterus bathybius*, *Sphyrna lewini* (scalloped hammerhead) and *Saurida undosquamis* (largescale saury, Fig 7A and 7B). *Pristipomoides multidens* was most abundant in Area 5 at sites with gravel and rubble habitat, while *Carangoides caeruleopinnatus* and *Lutjanus sebae* (red emperor) were associated with boulder/reef habitat. The presence of benthic biota (as bryozoans/encrusting animals, gorgonians, sponges, soft corals, hydroids and whips) was associated with higher numbers of *Gymnocranius grandoculis* (Robinson’s sea bream) and *Pristipomoides typus* (sharptooth snapper complex, Fig 7).

**Fig 8:**
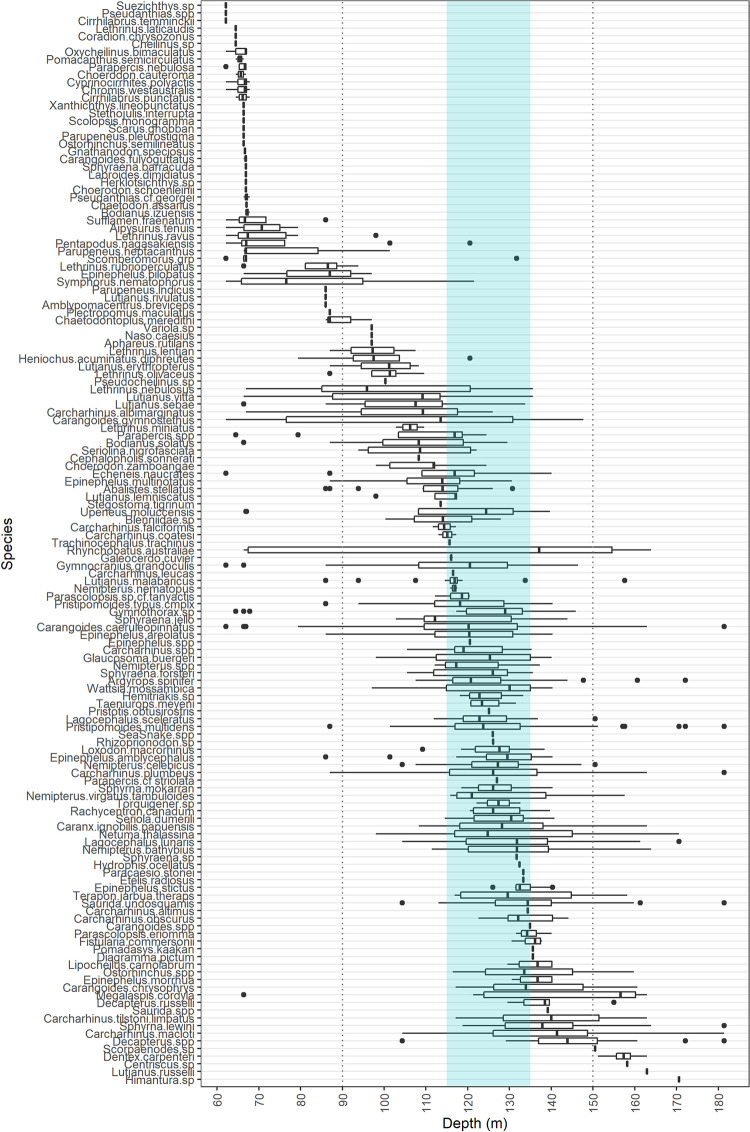
Boxplot of recorded species depth distributions ordered by mean depth. Middle lines indicate median depths, whiskers represent ~95% confidence intervals for comparing medians, and points are outliers. Depth range of the AC125 designation is indicated by blue shading.

Depth distributions identified a proportion of species prevalent at particular depths, with some only recorded on AC125 deployments (Fig 8). Of the 19 elasmobranch species recorded (6% of observations), two were observed only on the AC125, a *Stegostoma tigrinum* at 113.5 m (Area 2), and a *Carcharhinus leucas* at 116.5 m (Area 5).

A proportion of species were only prevalent at depths <70 m (e.g. *Oxychelinus bimaculatus*, *Pomacanthus semicirculatus*, *Choerodon cauteroma*, species from the genera *Cirrhilabrus*) or depths >150 m (e.g. *Dentex carpenteri*). Most other species were observed between 100 m and 150 m, with some across a broad depth range (e.g. *Rhynchobatus australiae*, whitespotted guitarfish, occurred between 66.4 and 163.9 m deep, Fig 8). Some species were recorded at depths deeper than previously reported. For example, *Stegostoma tigrinum* (zebra shark) has previously been recorded to 102 m [36] but occurred at 113.5 m in this study. Multiple *Rhynchobatus australiae* were recorded between 137.1 and 163.9 m, deeper than 128.8 m reported by Bond et al. [71]. Maximum depths of 135.6 m for *Pomadasys kaakan* (117 m: [72]), 181.4 m for *C*. *caeruleopinnatus* (129 m: [73]), and 162.9 m for *Lutjanus russelli* (131.7 m: [71]) are deeper than previous published records for the species (Fig 8). Species mobility is reflected by their depth distributions, with many ranging across the AC125 contour (shaded, Fig 8).

### Spatial patterns in fish species richness

Depth and hypsometric index (a surrogate for habitat complexity) calculated at large (hyp50) and intermediate (hyp25) window sizes, as well as the standard deviation of depth, were top predictors of fish species richness (S2 Fig, left). The common pattern of decline in richness with increasing depth and decreasing seafloor complexity supports GAMM models (S2 Fig, right). The fitted RF model for AC125 explained 26.18% of variance with RMSD = 3.06 between predicted and test data and Spearman rank correlation ρ = 0.42 (*p* < 0.001).

**Fig 9:**
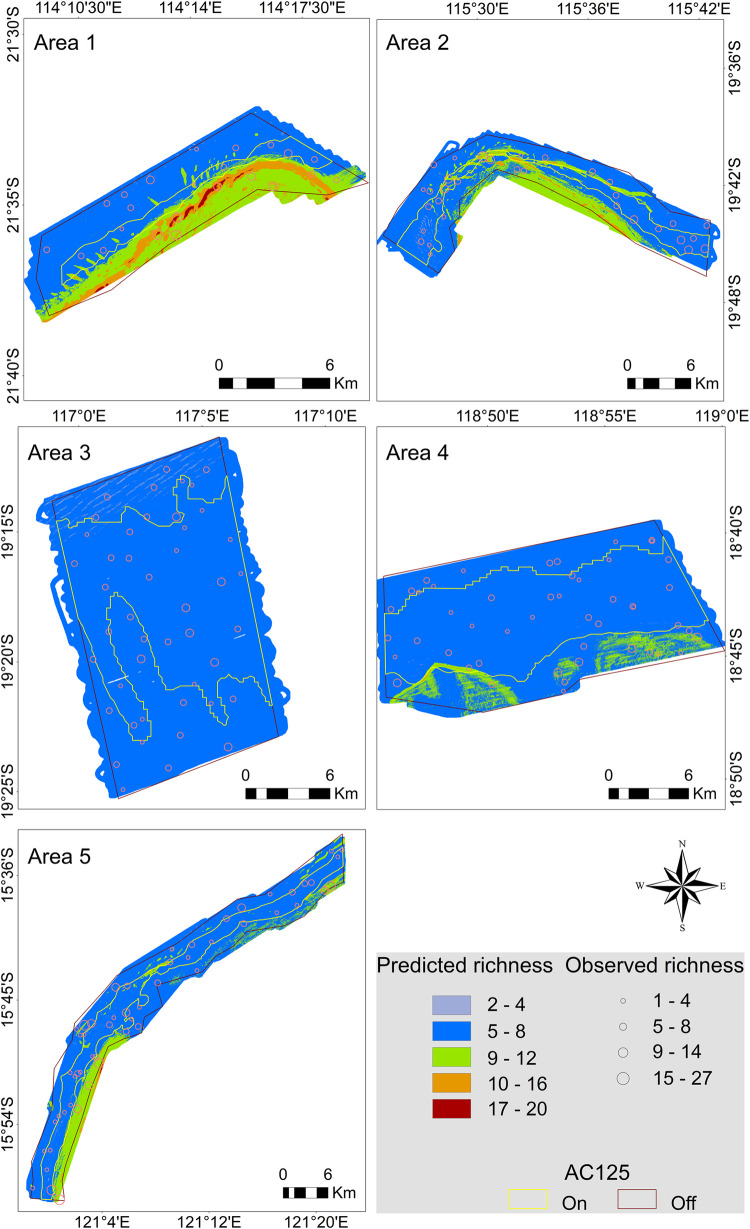
Predicted fish species richness across five AC125 study areas. Bubble size reflects the observed number of fish species in BRUVS deployments.

Higher species richness was predicted at shallower depths of all surveyed AC125 Areas except Area 3 which was predicted to have similar species richness throughout the area (Fig 9). Area 3 was the Area with the smallest depth range (36 m over approximately 20 km of seafloor; Table 3). Higher species richness was typically predicted outside of the designated AC125 with pockets of intermediate to high richness predicted to be spread throughout AC125 Areas 1, 2 and to a lesser extent in Area 5. In addition, a pocket of intermediate fish richness was predicted in the southern end of the AC125 in Area 4 (Fig 9).

### Regional spatial patterns in fish species richness

Depth was the clear predictor driving regional species richness, followed by habitat complexity at intermediate (hyp25) and large (hyp50) scales and aspect (asp) (S3 Fig). This mirrors the pattern observed with the AC125-specific richness model with an addition of direction of reef slope (aspect), where north and north-east facing reef slopes were predicted to have a higher fish richness. In contrast to the AC125 model, the regional model performed well with explained deviance of the fitted model of 65.33%. Despite having higher uncertainty (RMSD = 6.51), the Spearman rank correlation between predicted and test data was relatively high, *ρ* = 0.74 (*p* < 0.0001). The higher RMSD in the regional model was most probably caused by a high variance in the observed fish richness (1–74 species) on individual BRUVS samples in comparison to the AC125 model (observed species richness 1–27 species) which is known to affect model performance [74].

**Fig 10:**
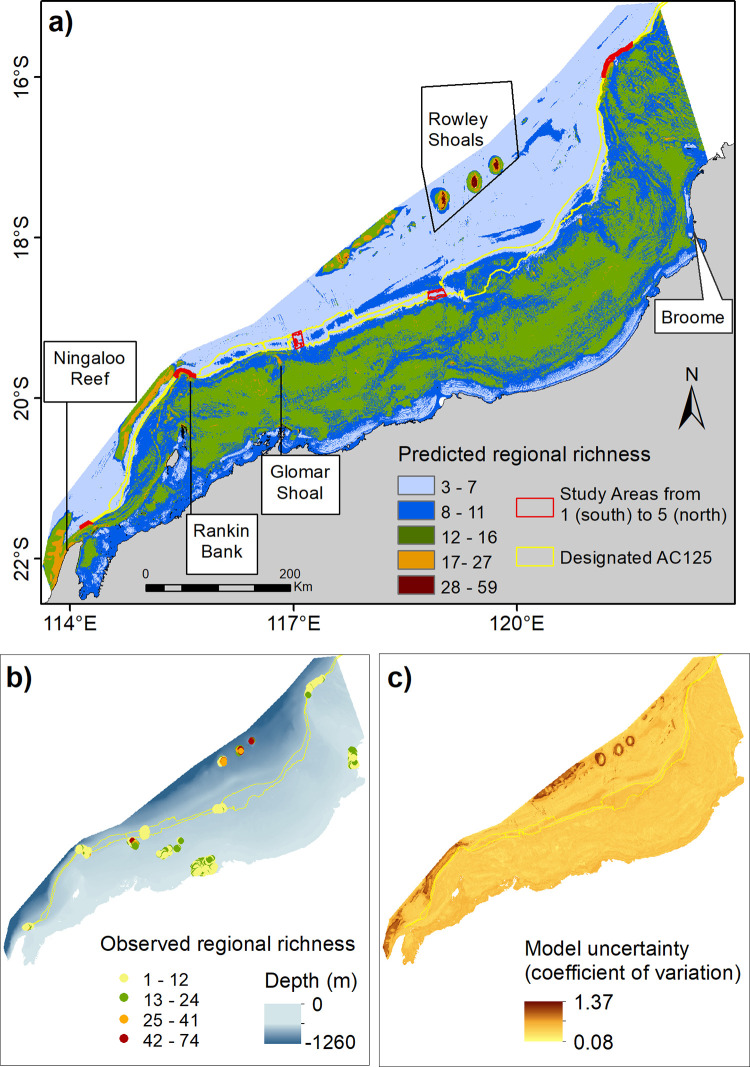
Predicted regional fish species richness for the North West Shelf (a) with locations of BRUVS deployments used in model fitting procedures plotted (b, bubble colours indicate the observed species richness) and associated uncertainty surface of the fitted RF model (c). This map was generated using ESRI software.

The regional map of predicted species richness indicated that AC125 Areas 1, 2 and 5 had intermediate richness between 8 and 27 species, similar species richness as was predicted for the north of Ningaloo Reef and the inner shelf across the greater NW region (Fig 10A). In contrast, Areas 3 and 4 had relatively low species richness (up to seven species), more representative of the outer shelf and nearshore locations. Pockets of high species richness were predicted throughout the region. These were mainly associated with offshore reefs, shoals, and banks (e.g. Rowley Shoals, Glomar Shoal, Rankin Bank; Fig 10A). The uncertainty of the predicted spatial model was generally low, except in the Areas with a wide range of observed species richness or for Areas where no field observations were collected (Fig 10B and 10C). Higher RMSD in the unsurveyed Areas further highlighted the importance of spatial coverage of samples for model training to suit the extent of the study area and to adequately represent natural variability in the study system in order to be able to predict the model in space or time [75].

## Discussion

Paleoshorelines, such as the designated AC125, and other geomorphic features shape biodiversity in north-western Australia [21, 22, 26, 76, 77], and improving our understanding and expanding the knowledge of the biodiversity in these systems is essential for effective management and conservation. The AC125 feature was thought to be structurally complex, supporting high biodiversity relative to surrounding areas. However, habitat complexity (substrate and benthos cover) was not higher at the surveyed sites on the AC125 (specifically at 115–135 m deep along this feature), and fish species richness and abundance were not greater than at sites immediately adjacent to the AC125. In fact, depth and structural complexity of the seafloor were the main drivers of fish species richness, on and off the AC125, as well as across the region. Fish diversity was greatest at shallow depths off the AC125 (76% of fish species overall), however, the AC125 does support a diverse fish assemblage, including ~53% of mesophotic fish species observed in this study as well as 39% of the recorded fish abundance. This indicates the fish fauna encompassed within the Key Ecological Feature, combined with the immediately adjacent habitats, are broadly representative of the mesophotic fish communities within the north west region, including species of commercial and conservation importance.

### Regional context of the AC125

The north-west region of Australia comprises a range of habitat types from shallow coral-reef habitats to deep abyssal plains [20]. Unsurprisingly, our results show that the AC125 fishes are less diverse than shallow reef regions, yet fish communities were similar to those of other mesophotic areas on the NWS. Most mesophotic research has typically focused on structurally complex mesophotic reef ecosystems, such as banks or shoals, that are known to support diverse communities of reef-associated fishes in relatively clear water [12, 78], which contrasts with the vast areas lacking hard substrate and higher turbidity that we observed during the AC125 surveys. Thus, our study is an important contribution to understanding fish diversity in less well-known marginal environments and in contextualising AC125 communities regionally.

Despite an overall lack of habitat complexity of the AC125 and adjacent areas spanning 60–140 m, fish diversity was comparable to other mesophotic areas on the NWS. For example, we identified 75 fish species/groups on the AC125, and 141 species overall; another recent study in the nearby Oceanic Shoals Marine Park recorded 103 species in 60–120 m depth (McLean et al. in prep). Diversity was also similar to the unique sand and gravel seascape of another KEF, Glomar Shoal (118 species at 60–78 m depth: [21, 38]), as well as to the areas of continental shelf offshore from Ningaloo Reef (an average of 10–15 species per deployment in 50–90 m depth [35], similar to the 14 species/deployment in the shallow off-AC125 sites. Inshore from Area 2, 2017 trawl surveys in the Montebello Marine Park identified 84 species at 60–70 m depth [30]. Thus, the mesophotic depths surveyed around and including the AC125 clearly support a representative number of species and comprise similar fish diversity compared to other regions on the NWS. In fact, the AC125 could be considered a transition zone between the continental shelf and continental rise and it is the combination of these features that support a diversity of fishes.

There was no evidence of a latitudinal gradient in fish community composition along the AC125, despite the fact our study spanned 1000 km across six degrees of latitude. Area along the AC125 was influential on fish community composition, but this was driven by the fact that Area 5 was distinct from all other areas, rather than associated with a change in fish community structure or composition from north to south. This is consistent with geological data that suggests the designated AC125 north of 17˚S comprises a different seascape composition to those areas south of 17˚S [21], with high gravel and low mud content. It is further supported by our results that show geomorphology and habitat, including depth, cover of soft sediment and substrate complexity were the most important drivers of fish community structure across the length of the AC125.

### Drivers of fish community composition on the AC125 and adjacent habitats

Species richness was not higher on the AC125 compared to adjacent habitats and the AC125 is typically located on the edge of the highest species richness zone. In all areas except Area 2, community analyses and spatial models indicated that average species richness (and abundance) was lower on the AC125 than in adjacent shallower sites off the AC125, and higher on the AC125 than in adjacent deeper sites off the AC125. These findings are consistent with patterns of decline in richness with increasing depth reported elsewhere across tropical marine taxa [1, 11, 79–81]. In Area 2, a greater number of species and individuals was observed on the AC125 than off the AC125, which is likely linked to its proximity to the biodiverse Rankin Bank. Deep sites at Rankin Bank (>40 m) support diverse fishes associated with high seabed rugosity, low proportions of sand substrate and presence of filter-feeding sponge biota [38]. Likewise, Area 2 was characterised by moderate depths (average 135 m) with complex habitat and benthic biota at some sites. While this represents pockets of higher local richness associated with the AC125, few sections of hard substrate were identified elsewhere on the AC125 escarpment. The original coastline feature is likely to have been buried by deposition of soft sediment, and indeed the sediments of the AC125 are of marine rather than terrestrial origin (Wakeford et al. In prep.). Deposition of marine sediments including sand and gravel [22] on sections of the AC125 is possible, as this feature would have experienced several episodes of sea-level fluctuation since the last glacial maximum approximately 20,000 years ago [26].

Habitat complexity was also an important driver of fish diversity, with more diverse and abundant fish communities associated with hard, structurally complex habitats, as has been reported by other studies [3, 36, 82]. The high species richness and abundance of fishes at some sites can be attributed to the occurrence of benthic biota such as bryozoans, gorgonians, hydroids and other filter-feeding organisms, which are common at mesophotic depths [1, 83] and were sparsely distributed in depths of 60–120 m across our study areas. Further, sites typically devoid of epibenthos were comprised of mud or silt and were usually characterised by low structural complexity and fewer fish. Richness was highest at shallow sites off AC125 in Area 1 (located adjacent to Ningaloo Reef at the most southern location) and Area 5 (the furthest north). High species richness is commonly observed in complex habitats at shallower depths [e.g. 84], and Areas 1 and 5 were the shallowest Areas sampled overall with higher proportions of complex habitat in shallow depths than for other Areas. Since Areas 1 and 5 were also relatively narrow in comparison to Areas 3 and 4 (4–7 km vs 21 and 12 km width), the relatively rapid change in depth is likely linked to steepness of the slope and structural complexity of the seabed. A broad range of depths (60–160 m) and thus multiple habitats were sampled in Area 1, from some of the deepest deployments on soft sediment, to gravel and rubble substrate, and structurally complex seabed with filter-feeding organisms in depths between 60 and 100 m. Also, a greater proportion of complex benthic biota was evident in Area 1, likely related to proximity to Ningaloo Reef. There was low diversity in Areas 3 and 4 due to small depth gradient, with comparably more soft-sediment substrate with low structural habitat complexity and benthos.

### Biodiversity features of the AC125 and adjacent habitats

Most species observed were typical of the region and habitats, and included some teleosts and elasmobranchs important to fisheries or of conservation concern that were sighted along the AC125 and in adjacent habitats. For example, *P*. *multidens* was the most abundant species observed in BRUVS on the AC125 overall (MaxN = 301), was associated with mid-depths on seabed with greater proportions of gravel and rubble substrate, and our models found it was most abundant in sites from the most northerly Area 5. This species is the primary target of the $12Mpa finfish fishery in Western Australia [39, 40], and is comprised of separate stocks along the NWS [40], so minimizing impacts to this species through management of the designated AC125 or Area 5 gravel/habitat will likely have flow-on benefits for the fishery.

Furthermore, we observed a total of 170 sharks and rays from 19 species in 60% of BRUVS deployments, including several elasmobranchs of conservation significance on and off the AC125. The bull shark (*C*. *leucas*, near threatened [85]) and zebra shark (*Stegostoma tigrinum*, endangered globally but of least concern in Australia, [86]) were observed only on the AC125. Yet mobility (*C*. *leucas* uses multiple habitats and *S*. *tigrinum* travels long distances seasonally [86]) suggests these elasmobranchs occur across a broad area. *Rhynchobatus australiae* [87, 88], *Sphyrna lewini* [89] and *S*. *mokarran* [90], which are critically endangered, were observed on and off the AC125. Generally, less is known about the population status of these elasmobranchs and declines are related to fishing and the value of fins in Southeast Asia. Australia’s low fishing pressure on wedgefishes represents a potential refuge or lifeboat for *R*. *australiae* [87] and potentially for other species that are more heavily fished and poorly managed elsewhere in their range. *Epinephelus stictus* (IUCN listed as of least concern, but of fishery importance in Asia) was observed only on the AC125 and was likely associated with the AC125 expanses of soft substrate, its preferred habitat [91, 92].

Most species displayed broad depth distributions which included the AC125, suggesting that many species have flexibility in vertical distribution. Certainly, we observed several species at depths beyond their known range, including *R*. *australiae*, *Stegostoma tigrinum*, *Lutjanus russelli* and *Pomadasys kaakan* which were all observed deeper than previously recorded in published literature [35, 70, 71, 92, 93]. Of note is that many species observed on and along the AC125 were mobile, predatory and use multiple habitats (e.g. roving sharks and snappers [94]), rather than the typical site-attached fishes of shallow coral reefs.

## Conclusions and recommendations

The designated AC125 was previously thought to comprise extensive areas of hard substrate and thus sites for high species diversity and enhanced species richness relative to surrounding areas of predominantly soft sediment [22]. We have shown that the AC125 does not support higher diversity than adjacent areas, but that the fish communities of the AC125 and adjacent habitats are representative of mesophotic depths of the NWS. Highest fish diverstiy and abundance was associated with complex habitat structure in isolated pockets along the AC125, but these were uncommon and most of the surveyed feature is characterised by soft sediment associated, and highly mobile, fish species. The surveyed portion of the AC125 appears to represent a transition zone between the continental shelf and continental rise fish communities, and as such management actions that conserve the designated AC125 will protect habitats of a large proportion of the mesophotic fish diversity of the NWS region, with likely flow-on effects for biodiversity, resilience, and fisheries stocks. However, to protect habitats of a greater diversity of fishes would require management actions that extend beyond the designated AC125 and that encompassed a greater variety of depths and habitats. For example, extending the KEF to include certain shallow (to 60 m deep) sections of high habitat complexity (i.e. large features such as the shelf and part of the rise [22]) would better encompass both soft-sediment and structurally complex habitats for fishes and hence habitats of a more representative proportion of mesophotic fishes on the NWS would be protected. Coupled with other fish management measures applied in the region (e.g. fisheries quotas and marine parks), protection of targeted additional habitats in the designated AC125 would achieve greatest fish biodiversity conservation outcomes across the region.

While further research is required to comprehensively document the species inhabiting the full length of the designated AC125, the breadth of our study spanning 1000km of the feature has provided a greatly expanded knowledge of mesophotic communities of the KEF and the NWS more generally. This research identified that fish communities were more influenced by factors other than the AC125, however further exploration is needed to recognize whether the AC125 plays an important role for other biota, such as megafauna. One of the challenges in managing Australia’s marine estate is its vast size, and the relative paucity of data, particularly in and beyond mesophotic depths. The costs and logistics associated with collecting rigorous data to provide comprehensive baseline knowledge and inform future monitoring efforts for the entire EEZ are insurmountable. Here we have shown the utility of approaches such as predictive spatial modelling for extrapolating results from targeted field surveys across the lengths of the AC125 feature and to enable regional comparisons. Such approaches will be invaluable for filling knowledge gaps in the future [95], and to inform effective management.

## Supporting information

S1 Fig Substrate and benthos at each area along the designated AC125. Hard complex substrate (boulder/reef and rubble) were highest in Areas 1 and 5, which also comprised the highest abundance of benthic biota (n refers to the number of BRUVS deployments).
(DOCX)

S2 Fig Variable importance (mean decrease in accuracy), left and partial response plots for top four predictors (values of the partial dependence function along Y-axis), right from the AC125 fish richness RF model. See Table 1 for predictor definitions.
(DOCX)

S3 Fig Variable importance (mean decrease in accuracy), left, and partial response plots for top four predictors (values of the partial dependence function along Y-axis) right, from the regional fish richness RF model. See Table 1 for predictor definitions.
(DOCX)

S1 Table Abundance and occurrence of each recorded family, genus, species/group by area and AC125 position. (DOCX)
