# Supplementary material for: Mesophotic fish communities of the ancient coastline in Western Australia
Source: PLoS One. 2021 Apr 21;16(4):e0250427. doi: 10.1371/journal.pone.0250427 (PMC8059809; doi:10.1371/journal.pone.0250427)
Supplement: S1 Fig — Hard complex substrate (boulder/reef and rubble) were highest in Areas 1 and 5, which also comprised the highest abundance of benthic biota (n refers to the number of BRUVS deployments). (DOCX) [file pone.0250427.s001.docx]

Mesophotic fish communities of the ancient coastline in Western Australia

Leanne M. Currey-Randall^1*^, Ronen Galaiduk^2^, Marcus Stowar^1^, Brigit I. Vaughan^2^, Karen J. Miller^2^

^1^Australian Institute of Marine Science, Townsville, Queensland, Australia

^2^Australian Institute of Marine Science, Indian Ocean Marine Research Centre, University of Western Australia, Crawley, Western Australia, Australia

* Corresponding author

E-mail: l.currey@aims.gov.au (LMCR)


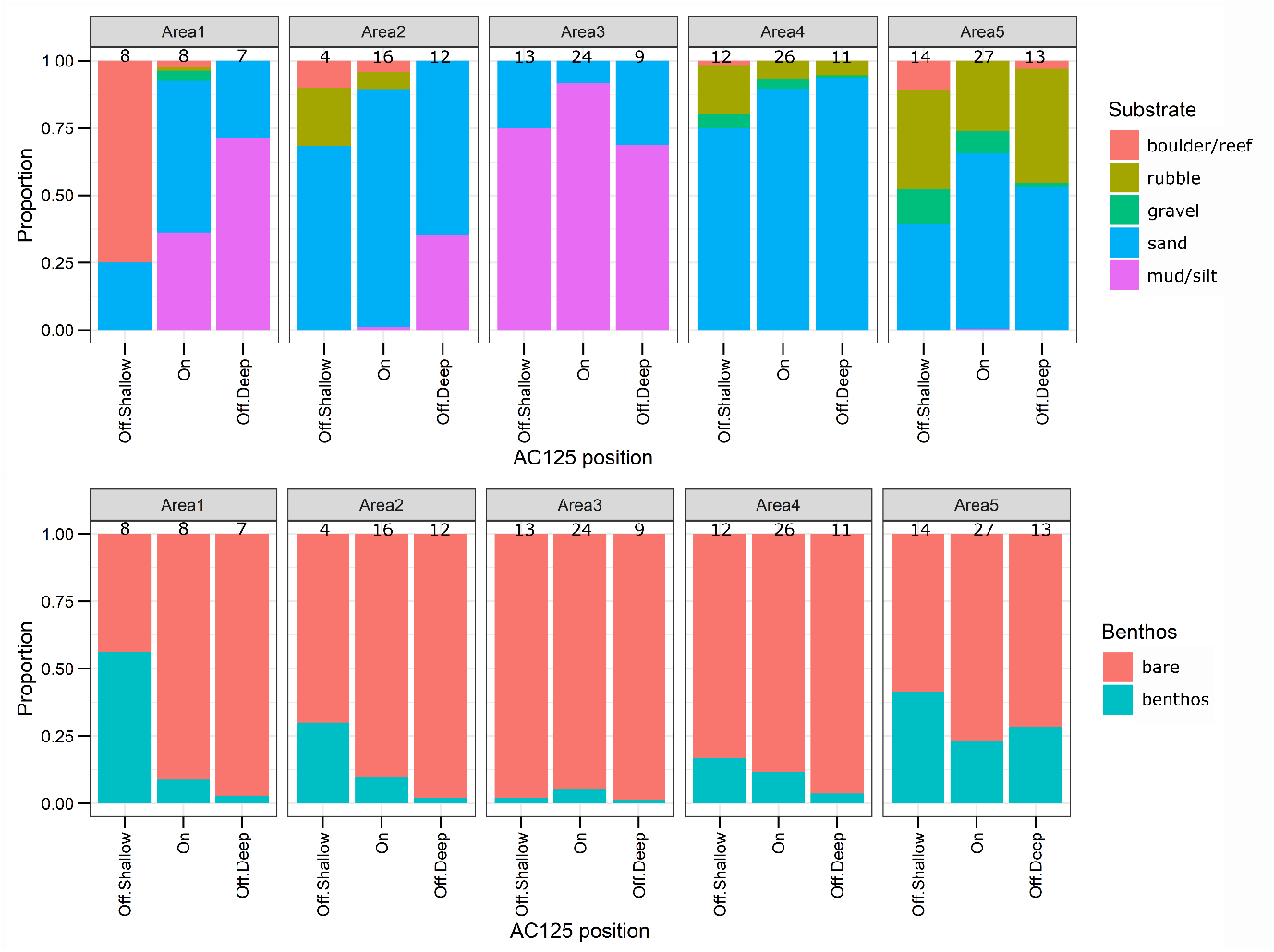


S1 Fig. Substrate and benthos at each Area along the AC125. Hard complex substrate (boulder/reef and rubble) were highest in Areas 1 and 5, which also comprised the highest abundance of benthic biota (n refers to the number of BRUVS deployments).
