# Supplementary material for: Mesophotic fish communities of the ancient coastline in Western Australia
Source: PLoS One. 2021 Apr 21;16(4):e0250427. doi: 10.1371/journal.pone.0250427 (PMC8059809; doi:10.1371/journal.pone.0250427)
Supplement: S3 Fig — See Table 1 for predictor definitions. (DOCX) [file pone.0250427.s003.docx]

Mesophotic fish communities of the ancient coastline in Western Australia

Leanne M. Currey-Randall^1*^, Ronen Galaiduk^2^, Marcus Stowar^1^, Brigit I. Vaughan^2^, Karen J. Miller^2^

^1^Australian Institute of Marine Science, Townsville, Queensland, Australia

^2^Australian Institute of Marine Science, Indian Ocean Marine Research Centre, University of Western Australia, Crawley, Western Australia, Australia

* Corresponding author

E-mail: l.currey@aims.gov.au (LMCR)


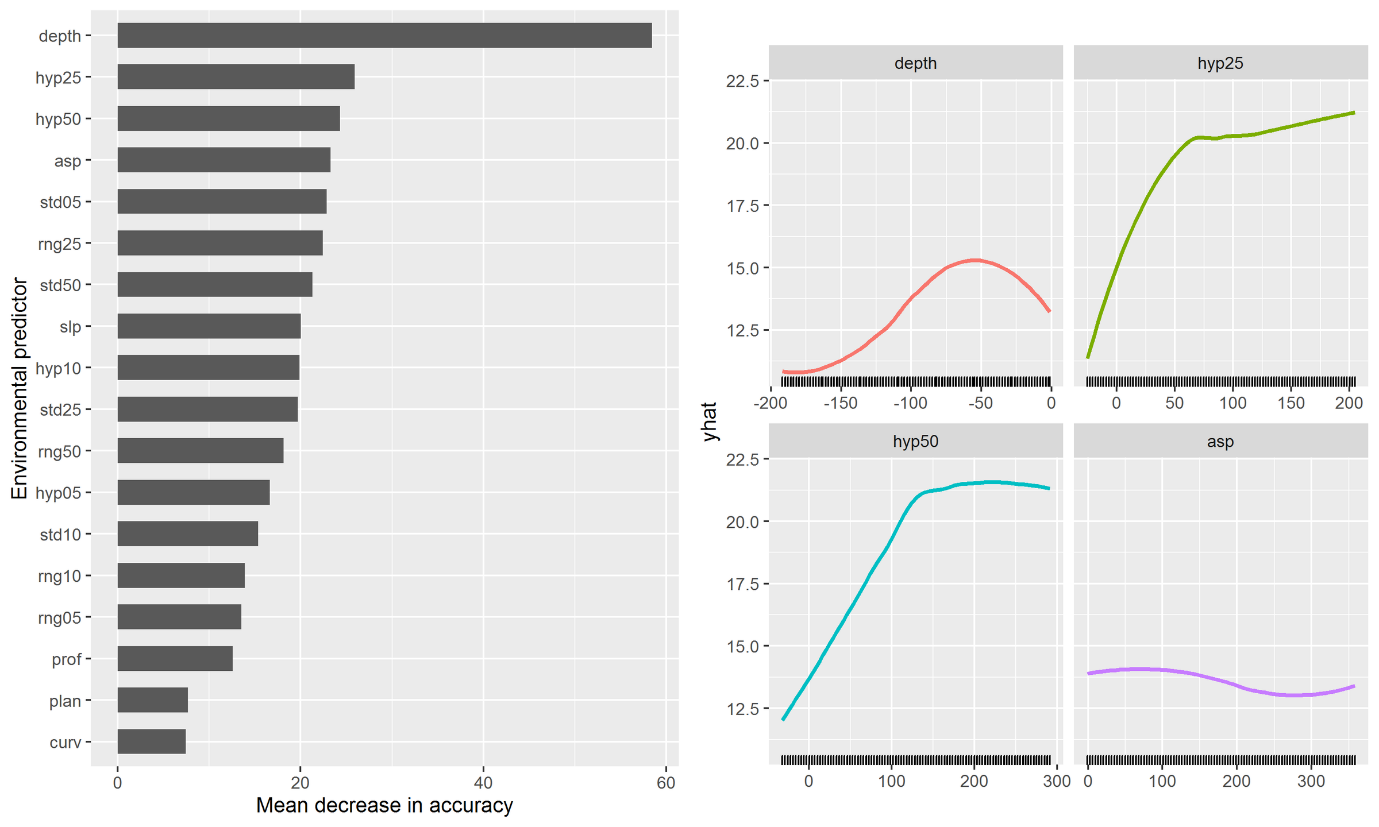
S3 Fig. Variable importance (mean decrease in accuracy, left), and partial response plots for top four predictors (values of the partial dependence function along Y-axis, right), from the regional fish richness RF model. See Table 1 for predictor definitions.
