# Supplementary material for: Mesophotic fish communities of the ancient coastline in Western Australia
Source: PLoS One. 2021 Apr 21;16(4):e0250427. doi: 10.1371/journal.pone.0250427 (PMC8059809; doi:10.1371/journal.pone.0250427)
Supplement: S1 Table — (DOCX) [file pone.0250427.s004.docx]

Mesophotic fish communities of the ancient coastline in Western Australia

Leanne M. Currey-Randall^1*^, Ronen Galaiduk^2^, Marcus Stowar^1^, Brigit I. Vaughan^2^, Karen J. Miller^2^

^1^Australian Institute of Marine Science, Townsville, Queensland, Australia

^2^Australian Institute of Marine Science, Indian Ocean Marine Research Centre, University of Western Australia, Crawley, Western Australia, Australia

* Corresponding author

E-mail: l.currey@aims.gov.au (LMCR)

S1 Table. Abundance and occurrence of each recorded family, genus, species/group by Area and AC125 position.

|  | Area 1 | | | Area 2 | | | Area 3 | | | Area 4 | | | Area 5 | | |  |
| --- | --- | --- | --- | --- | --- | --- | --- | --- | --- | --- | --- | --- | --- | --- | --- | --- |
|  | Off Shallow | On | Off Deep | Off Shallow | On | Off Deep | Off Shallow | On | Off Deep | Off Shallow | On | Off Deep | Off Shallow | On | Off Deep | Total |
| Species / n BRUVS | 8 | 8 | 7 | 4 | 16 | 12 | 13 | 24 | 9 | 12 | 26 | 11 | 14 | 27 | 13 | 204 |
| *Abalistes stellatus* | 0 | 0 | 0 | 2 | 2 | 1 | 2 | 0 | 0 | 2 | 0 | 0 | 6 | 3 | 0 | 18 |
| *Aipysurus tenuis* | 2 | 0 | 0 | 0 | 0 | 0 | 0 | 0 | 0 | 0 | 0 | 0 | 0 | 0 | 0 | 2 |
| *Amblypomacentrus breviceps* | 0 | 0 | 0 | 0 | 0 | 0 | 0 | 0 | 0 | 0 | 0 | 0 | 1 | 0 | 0 | 1 |
| *Aphareus rutilans* | 0 | 0 | 0 | 0 | 0 | 0 | 0 | 0 | 0 | 0 | 0 | 0 | 1 | 0 | 0 | 1 |
| *Argyrops spinifer* | 0 | 5 | 1 | 2 | 28 | 8 | 13 | 20 | 0 | 12 | 7 | 1 | 2 | 0 | 0 | 99 |
| *Blenniidae* sp | 0 | 0 | 0 | 1 | 1 | 0 | 0 | 0 | 0 | 0 | 0 | 0 | 0 | 0 | 0 | 2 |
| *Bodianus izuensis* | 5 | 0 | 0 | 0 | 0 | 0 | 0 | 0 | 0 | 0 | 0 | 0 | 0 | 0 | 0 | 5 |
| *Bodianus solatus* | 1 | 0 | 0 | 0 | 2 | 0 | 0 | 0 | 0 | 2 | 0 | 0 | 4 | 2 | 1 | 12 |
| *Carangoides caeruleopinnatus* | 5 | 17 | 3 | 10 | 61 | 34 | 8 | 16 | 3 | 14 | 16 | 24 | 28 | 2 | 0 | 241 |
| *Carangoides chrysophrys* | 0 | 3 | 2 | 0 | 5 | 3 | 1 | 0 | 0 | 0 | 1 | 0 | 0 | 1 | 0 | 16 |
| *Carangoides fulvoguttatus* | 11 | 0 | 0 | 0 | 0 | 0 | 0 | 0 | 0 | 0 | 0 | 0 | 0 | 0 | 0 | 11 |
| *Carangoides gymnostethus* | 9 | 0 | 0 | 0 | 5 | 12 | 0 | 0 | 0 | 0 | 1 | 0 | 12 | 6 | 0 | 45 |
| *Carangoides* spp | 0 | 0 | 0 | 0 | 0 | 0 | 0 | 0 | 0 | 0 | 1 | 0 | 0 | 0 | 0 | 1 |
| *Caranx ignonilis/ papuensis* | 0 | 0 | 1 | 0 | 2 | 3 | 12 | 4 | 3 | 2 | 5 | 4 | 0 | 0 | 0 | 36 |
| *Carcharhinus albimarginatus* | 1 | 0 | 0 | 1 | 1 | 0 | 0 | 0 | 0 | 0 | 0 | 0 | 4 | 1 | 0 | 8 |
| *Carcharhinus altimus* | 0 | 0 | 0 | 0 | 0 | 0 | 0 | 0 | 2 | 0 | 0 | 0 | 0 | 0 | 0 | 2 |
| *Carcharhinus coatesi* | 0 | 0 | 0 | 0 | 0 | 0 | 2 | 0 | 0 | 0 | 0 | 0 | 0 | 0 | 0 | 2 |
| *Carcharhinus falciformis* | 0 | 0 | 0 | 0 | 0 | 0 | 1 | 0 | 0 | 1 | 0 | 0 | 0 | 0 | 0 | 2 |
| *Carcharhinus leucas* | 0 | 0 | 0 | 0 | 0 | 0 | 0 | 0 | 0 | 0 | 0 | 0 | 0 | 1 | 0 | 1 |
| *Carcharhinus macloti* | 2 | 3 | 6 | 0 | 5 | 8 | 0 | 1 | 3 | 0 | 2 | 4 | 0 | 0 | 0 | 34 |
| *Carcharhinus obscurus* | 0 | 0 | 0 | 0 | 0 | 0 | 0 | 3 | 4 | 0 | 3 | 1 | 0 | 0 | 0 | 11 |
| *Carcharhinus plumbeus* | 0 | 0 | 0 | 0 | 1 | 2 | 0 | 0 | 1 | 1 | 4 | 1 | 8 | 13 | 6 | 37 |
| *Carcharhinus* spp | 0 | 0 | 0 | 0 | 0 | 0 | 2 | 8 | 0 | 1 | 2 | 0 | 0 | 0 | 0 | 13 |
| *Carcharhinus tilstoni/ limbatus* | 0 | 0 | 0 | 0 | 0 | 1 | 1 | 0 | 0 | 0 | 0 | 0 | 0 | 0 | 0 | 2 |
| *Centriscus* sp | 0 | 0 | 1 | 0 | 0 | 0 | 0 | 0 | 0 | 0 | 0 | 0 | 0 | 0 | 0 | 1 |
| *Cephalopholis sonnerati* | 0 | 0 | 0 | 0 | 0 | 0 | 0 | 0 | 0 | 1 | 0 | 0 | 0 | 0 | 0 | 1 |
| *Chaetodon assarius* | 1 | 0 | 0 | 0 | 0 | 0 | 0 | 0 | 0 | 0 | 0 | 0 | 0 | 0 | 0 | 1 |
| *Chaetodontoplus meredithi* | 0 | 0 | 0 | 0 | 0 | 0 | 0 | 0 | 0 | 0 | 0 | 0 | 4 | 0 | 0 | 4 |
| *Cheilinus* sp | 1 | 0 | 0 | 0 | 0 | 0 | 0 | 0 | 0 | 0 | 0 | 0 | 0 | 0 | 0 | 1 |
| *Choerodon cauteroma* | 2 | 0 | 0 | 0 | 0 | 0 | 0 | 0 | 0 | 0 | 0 | 0 | 0 | 0 | 0 | 2 |
| *Choerodon schoenleinii* | 1 | 0 | 0 | 0 | 0 | 0 | 0 | 0 | 0 | 0 | 0 | 0 | 0 | 0 | 0 | 1 |
| *Choerodon zamboangae* | 0 | 0 | 0 | 0 | 0 | 0 | 0 | 0 | 0 | 1 | 0 | 0 | 3 | 1 | 0 | 5 |
| *Chromis westaustralis* | 33 | 0 | 0 | 0 | 0 | 0 | 0 | 0 | 0 | 0 | 0 | 0 | 0 | 0 | 0 | 33 |
| *Cirrhilabrus punctatus* | 3 | 0 | 0 | 0 | 0 | 0 | 0 | 0 | 0 | 0 | 0 | 0 | 0 | 0 | 0 | 3 |
| *Cirrhilabrus temminckii* | 4 | 0 | 0 | 0 | 0 | 0 | 0 | 0 | 0 | 0 | 0 | 0 | 0 | 0 | 0 | 4 |
| *Coradion chrysozonus* | 1 | 0 | 0 | 0 | 0 | 0 | 0 | 0 | 0 | 0 | 0 | 0 | 0 | 0 | 0 | 1 |
| *Cyprinocirrhites polyactis* | 17 | 0 | 0 | 0 | 0 | 0 | 0 | 0 | 0 | 0 | 0 | 0 | 0 | 0 | 0 | 17 |
| *Decapterus russelli* | 0 | 2 | 3 | 0 | 0 | 0 | 0 | 0 | 0 | 0 | 11 | 11 | 0 | 0 | 0 | 27 |
| *Decapterus* spp | 1 | 0 | 0 | 0 | 7 | 35 | 0 | 0 | 0 | 0 | 8 | 3 | 0 | 0 | 0 | 54 |
| *Dentex carpenteri* | 0 | 0 | 0 | 0 | 0 | 4 | 0 | 0 | 0 | 0 | 0 | 0 | 0 | 0 | 2 | 6 |
| *Diagramma pictum* | 0 | 1 | 0 | 0 | 0 | 0 | 0 | 0 | 0 | 0 | 0 | 0 | 0 | 0 | 0 | 1 |
| *Echeneis naucrates* | 1 | 0 | 0 | 0 | 1 | 0 | 4 | 0 | 0 | 0 | 0 | 1 | 1 | 2 | 0 | 10 |
| *Epinephelus amblycephalus* | 0 | 1 | 0 | 0 | 1 | 0 | 0 | 0 | 0 | 0 | 0 | 0 | 3 | 13 | 10 | 28 |
| *Epinephelus areolatus* | 0 | 1 | 0 | 1 | 6 | 0 | 0 | 1 | 0 | 18 | 4 | 0 | 33 | 44 | 26 | 134 |
| *Epinephelus bilobatus* | 1 | 0 | 0 | 0 | 0 | 0 | 0 | 0 | 0 | 0 | 0 | 0 | 2 | 0 | 0 | 3 |
| *Epinephelus morrhua* | 0 | 0 | 0 | 0 | 0 | 0 | 0 | 0 | 0 | 0 | 0 | 0 | 0 | 1 | 3 | 4 |
| *Epinephelus multinotatus* | 0 | 0 | 0 | 0 | 2 | 0 | 0 | 0 | 0 | 2 | 0 | 0 | 4 | 2 | 0 | 10 |
| *Epinephelus* spp | 0 | 0 | 0 | 0 | 0 | 0 | 0 | 0 | 0 | 0 | 0 | 0 | 0 | 1 | 0 | 1 |
| *Epinephelus stictus* | 0 | 0 | 0 | 0 | 0 | 0 | 0 | 0 | 0 | 0 | 0 | 0 | 0 | 9 | 6 | 15 |
| *Etelis radiosus* | 0 | 0 | 0 | 0 | 0 | 0 | 0 | 0 | 0 | 0 | 0 | 0 | 0 | 0 | 2 | 2 |
| *Fistularia commersonii* | 0 | 0 | 0 | 0 | 0 | 0 | 0 | 0 | 0 | 0 | 2 | 0 | 0 | 0 | 2 | 4 |
| *Galeocerdo cuvier* | 0 | 0 | 0 | 0 | 0 | 0 | 1 | 0 | 0 | 0 | 0 | 0 | 1 | 0 | 0 | 2 |
| *Glaucosoma buergeri* | 0 | 0 | 0 | 0 | 0 | 0 | 0 | 0 | 0 | 0 | 0 | 0 | 1 | 2 | 2 | 5 |
| *Gnathanodon speciosus* | 2 | 0 | 0 | 0 | 0 | 0 | 0 | 0 | 0 | 0 | 0 | 0 | 0 | 0 | 0 | 2 |
| *Gymnocranius grandoculis* | 4 | 2 | 0 | 7 | 15 | 4 | 0 | 0 | 0 | 5 | 2 | 1 | 34 | 12 | 4 | 90 |
| *Gymnothorax* sp | 3 | 0 | 0 | 0 | 1 | 0 | 0 | 1 | 1 | 0 | 1 | 0 | 0 | 5 | 4 | 16 |
| *Hemitriakis* sp | 0 | 0 | 0 | 0 | 1 | 0 | 0 | 0 | 0 | 0 | 1 | 0 | 0 | 0 | 1 | 3 |
| *Heniochus acuminatus/ diphreutes* | 1 | 0 | 0 | 0 | 22 | 0 | 0 | 0 | 0 | 0 | 0 | 0 | 6 | 0 | 0 | 29 |
| *Herklotsichthys* sp | 4 | 0 | 0 | 0 | 0 | 0 | 0 | 0 | 0 | 0 | 0 | 0 | 0 | 0 | 0 | 4 |
| *Himantura* sp | 0 | 0 | 0 | 0 | 0 | 1 | 0 | 0 | 0 | 0 | 0 | 0 | 0 | 0 | 0 | 1 |
| *Hydrophis ocellatus* | 0 | 0 | 0 | 0 | 1 | 0 | 0 | 0 | 0 | 0 | 0 | 0 | 0 | 0 | 0 | 1 |
| *Labroides dimidiatus* | 2 | 0 | 0 | 0 | 0 | 0 | 0 | 0 | 0 | 0 | 0 | 0 | 0 | 0 | 0 | 2 |
| *Lagocephalus lunaris* | 2 | 3 | 5 | 0 | 3 | 4 | 11 | 8 | 14 | 11 | 54 | 28 | 0 | 0 | 0 | 143 |
| *Lagocephalus sceleratus* | 0 | 0 | 0 | 0 | 5 | 0 | 0 | 0 | 0 | 0 | 0 | 0 | 4 | 3 | 1 | 13 |
| *Lethrinus laticaudis* | 4 | 0 | 0 | 0 | 0 | 0 | 0 | 0 | 0 | 0 | 0 | 0 | 0 | 0 | 0 | 4 |
| *Lethrinus lentjan* | 0 | 0 | 0 | 0 | 0 | 0 | 0 | 0 | 0 | 2 | 0 | 0 | 4 | 0 | 0 | 6 |
| *Lethrinus miniatus* | 0 | 0 | 0 | 3 | 0 | 0 | 0 | 0 | 0 | 0 | 0 | 0 | 0 | 0 | 0 | 3 |
| *Lethrinus nebulosus* | 3 | 4 | 0 | 0 | 0 | 0 | 0 | 0 | 0 | 0 | 0 | 0 | 4 | 7 | 0 | 18 |
| *Lethrinus olivaceus* | 0 | 0 | 0 | 2 | 0 | 0 | 0 | 0 | 0 | 0 | 0 | 0 | 10 | 0 | 0 | 12 |
| *Lethrinus ravus* | 11 | 0 | 0 | 0 | 0 | 0 | 0 | 0 | 0 | 0 | 0 | 0 | 2 | 0 | 0 | 13 |
| *Lethrinus rubrioperculatus* | 4 | 0 | 0 | 0 | 0 | 0 | 0 | 0 | 0 | 0 | 0 | 0 | 12 | 0 | 0 | 16 |
| *Lipocheilus carnolabrum* | 0 | 0 | 0 | 0 | 0 | 0 | 0 | 0 | 0 | 0 | 0 | 0 | 0 | 0 | 4 | 4 |
| *Loxodon macrorhinus* | 0 | 1 | 0 | 0 | 3 | 0 | 0 | 2 | 0 | 1 | 5 | 2 | 0 | 0 | 1 | 15 |
| *Lutjanus erythropterus* | 0 | 0 | 0 | 0 | 0 | 0 | 0 | 0 | 0 | 61 | 0 | 0 | 7 | 0 | 0 | 68 |
| *Lutjanus lemniscatus* | 0 | 0 | 0 | 0 | 0 | 0 | 0 | 3 | 0 | 0 | 0 | 0 | 2 | 1 | 0 | 6 |
| *Lutjanus malabaricus* | 0 | 0 | 0 | 0 | 2 | 4 | 7 | 7 | 0 | 2 | 0 | 0 | 18 | 0 | 0 | 40 |
| *Lutjanus rivulatus* | 0 | 0 | 0 | 0 | 0 | 0 | 0 | 0 | 0 | 0 | 0 | 0 | 1 | 0 | 0 | 1 |
| *Lutjanus russelli* | 0 | 0 | 0 | 0 | 0 | 3 | 0 | 0 | 0 | 0 | 0 | 0 | 0 | 0 | 0 | 3 |
| *Lutjanus sebae* | 1 | 0 | 0 | 1 | 2 | 3 | 0 | 0 | 0 | 10 | 0 | 0 | 12 | 0 | 0 | 29 |
| *Lutjanus vitta* | 4 | 3 | 0 | 0 | 0 | 0 | 0 | 0 | 0 | 13 | 2 | 0 | 2 | 0 | 0 | 24 |
| *Megalaspis cordyla* | 2 | 10 | 20 | 0 | 1 | 1 | 0 | 0 | 0 | 0 | 0 | 0 | 0 | 0 | 0 | 34 |
| *Naso caesius* | 0 | 0 | 0 | 0 | 0 | 0 | 0 | 0 | 0 | 0 | 0 | 0 | 1 | 0 | 0 | 1 |
| *Nemipterus bathybius* | 0 | 4 | 14 | 0 | 1 | 2 | 48 | 40 | 32 | 1 | 55 | 15 | 5 | 12 | 9 | 238 |
| *Nemipterus celebicus* | 4 | 4 | 1 | 0 | 7 | 0 | 3 | 0 | 0 | 1 | 17 | 1 | 0 | 11 | 4 | 53 |
| *Nemipterus nematopus* | 0 | 0 | 0 | 0 | 0 | 0 | 2 | 1 | 0 | 0 | 0 | 0 | 0 | 0 | 0 | 3 |
| *Nemipterus* spp | 0 | 0 | 0 | 0 | 0 | 0 | 1 | 0 | 0 | 2 | 0 | 0 | 0 | 0 | 1 | 4 |
| *Nemipterus virgatus/ tambuloides* | 0 | 0 | 0 | 0 | 0 | 1 | 9 | 27 | 9 | 0 | 0 | 0 | 0 | 0 | 0 | 46 |
| *Netuma thalassina* | 0 | 1 | 8 | 0 | 6 | 11 | 11 | 10 | 1 | 15 | 2 | 4 | 1 | 0 | 0 | 70 |
| *Ostorhinchus semilineatus* | 4 | 0 | 0 | 0 | 0 | 0 | 0 | 0 | 0 | 0 | 0 | 0 | 0 | 0 | 0 | 4 |
| *Ostorhinchus* spp | 0 | 0 | 7 | 0 | 0 | 0 | 1 | 5 | 5 | 0 | 0 | 0 | 0 | 0 | 0 | 18 |
| *Oxycheilinus bimaculatus* | 5 | 0 | 0 | 0 | 0 | 0 | 0 | 0 | 0 | 0 | 0 | 0 | 0 | 0 | 0 | 5 |
| *Paracaesio stonei* | 0 | 0 | 0 | 0 | 0 | 0 | 0 | 0 | 0 | 0 | 0 | 0 | 0 | 0 | 8 | 8 |
| *Parapercis* cf *striolata* | 0 | 0 | 0 | 0 | 0 | 0 | 0 | 0 | 0 | 0 | 0 | 0 | 0 | 1 | 0 | 1 |
| *Parapercis nebulosa* | 6 | 0 | 0 | 0 | 0 | 0 | 0 | 0 | 0 | 0 | 0 | 0 | 0 | 0 | 0 | 6 |
| *Parapercis* spp | 3 | 0 | 0 | 0 | 0 | 0 | 0 | 0 | 0 | 0 | 1 | 0 | 3 | 4 | 0 | 11 |
| *Parascolopsis eriomma* | 0 | 0 | 0 | 0 | 0 | 0 | 0 | 0 | 0 | 0 | 0 | 0 | 0 | 2 | 7 | 9 |
| *Parascolopsis* cf *tanyactis* | 0 | 0 | 0 | 0 | 0 | 0 | 0 | 0 | 0 | 1 | 0 | 0 | 0 | 6 | 0 | 7 |
| *Parupeneus heptacanthus* | 2 | 0 | 0 | 0 | 0 | 0 | 0 | 0 | 0 | 0 | 0 | 0 | 1 | 0 | 0 | 3 |
| *Parupeneus indicus* | 0 | 0 | 0 | 0 | 0 | 0 | 0 | 0 | 0 | 0 | 0 | 0 | 1 | 0 | 0 | 1 |
| *Parupeneus pleurostigma* | 1 | 0 | 0 | 0 | 0 | 0 | 0 | 0 | 0 | 0 | 0 | 0 | 0 | 0 | 0 | 1 |
| *Pentapodus nagasakiensis* | 63 | 0 | 0 | 0 | 0 | 0 | 0 | 0 | 0 | 0 | 0 | 0 | 1 | 1 | 0 | 65 |
| *Plectropomus maculatus* | 0 | 0 | 0 | 0 | 0 | 0 | 0 | 0 | 0 | 0 | 0 | 0 | 1 | 0 | 0 | 1 |
| *Pomacanthus semicirculatus* | 2 | 0 | 0 | 0 | 0 | 0 | 0 | 0 | 0 | 0 | 0 | 0 | 0 | 0 | 0 | 2 |
| *Pomadasys kaakan* | 0 | 1 | 0 | 0 | 0 | 0 | 0 | 0 | 0 | 0 | 0 | 0 | 0 | 0 | 0 | 1 |
| *Pristipomoides multidens* | 0 | 8 | 1 | 2 | 21 | 11 | 30 | 53 | 3 | 27 | 12 | 7 | 30 | 49 | 47 | 301 |
| *Pristipomoides typus* complex | 0 | 0 | 0 | 11 | 9 | 2 | 0 | 6 | 0 | 11 | 4 | 0 | 24 | 37 | 27 | 131 |
| *Pristotis obtusirostris* | 0 | 0 | 0 | 0 | 0 | 0 | 0 | 0 | 0 | 0 | 0 | 0 | 0 | 1 | 0 | 1 |
| *Pseudanthias cf georgei* | 19 | 0 | 0 | 0 | 0 | 0 | 0 | 0 | 0 | 0 | 0 | 0 | 0 | 0 | 0 | 19 |
| *Pseudanthias* spp | 1 | 0 | 0 | 0 | 0 | 0 | 0 | 0 | 0 | 0 | 0 | 0 | 0 | 0 | 0 | 1 |
| *Pseudocheilinus* sp | 0 | 0 | 0 | 1 | 0 | 0 | 0 | 0 | 0 | 0 | 0 | 0 | 0 | 0 | 0 | 1 |
| *Rachycentron canadum* | 0 | 1 | 0 | 0 | 1 | 1 | 0 | 0 | 0 | 0 | 3 | 2 | 0 | 0 | 0 | 8 |
| *Rhizoprionodon* sp | 0 | 0 | 0 | 0 | 0 | 0 | 0 | 0 | 0 | 0 | 0 | 1 | 0 | 0 | 0 | 1 |
| *Rhynchobatus australiae* | 3 | 0 | 2 | 0 | 0 | 1 | 0 | 0 | 0 | 0 | 1 | 0 | 0 | 0 | 0 | 7 |
| *Saurida* spp | 0 | 0 | 0 | 0 | 0 | 0 | 0 | 0 | 0 | 0 | 1 | 0 | 0 | 0 | 0 | 1 |
| *Saurida undosquamis* | 1 | 1 | 4 | 0 | 2 | 3 | 4 | 17 | 12 | 0 | 20 | 13 | 0 | 4 | 0 | 81 |
| *Scarus ghobban* | 1 | 0 | 0 | 0 | 0 | 0 | 0 | 0 | 0 | 0 | 0 | 0 | 0 | 0 | 0 | 1 |
| *Scolopsis monogramma* | 1 | 0 | 0 | 0 | 0 | 0 | 0 | 0 | 0 | 0 | 0 | 0 | 0 | 0 | 0 | 1 |
| *Scomberomorus* grp | 5 | 0 | 0 | 0 | 0 | 0 | 0 | 0 | 1 | 0 | 0 | 0 | 0 | 0 | 0 | 6 |
| *Scorpaenodes* sp | 0 | 0 | 0 | 0 | 0 | 0 | 0 | 0 | 0 | 0 | 0 | 0 | 0 | 0 | 1 | 1 |
| Sea Snake unidenified | 0 | 0 | 0 | 0 | 1 | 0 | 0 | 0 | 0 | 0 | 0 | 0 | 0 | 0 | 0 | 1 |
| *Seriola dumerili* | 0 | 3 | 0 | 0 | 11 | 0 | 0 | 0 | 0 | 0 | 0 | 1 | 2 | 10 | 4 | 31 |
| *Seriolina nigrofasciata* | 0 | 0 | 0 | 0 | 1 | 0 | 0 | 0 | 1 | 0 | 0 | 0 | 2 | 0 | 0 | 4 |
| *Sphyraena barracuda* | 2 | 0 | 0 | 0 | 0 | 0 | 0 | 0 | 0 | 0 | 0 | 0 | 0 | 0 | 0 | 2 |
| *Sphyraena forsteri* | 0 | 2 | 0 | 0 | 4 | 0 | 0 | 0 | 0 | 25 | 0 | 0 | 1 | 0 | 0 | 32 |
| *Sphyraena jello* | 0 | 1 | 1 | 2 | 0 | 0 | 0 | 0 | 0 | 7 | 0 | 0 | 0 | 0 | 0 | 11 |
| *Sphyraena* sp | 0 | 0 | 0 | 0 | 0 | 0 | 0 | 0 | 1 | 0 | 0 | 0 | 0 | 0 | 0 | 1 |
| *Sphyrna lewini* | 0 | 0 | 1 | 0 | 0 | 2 | 0 | 8 | 3 | 0 | 0 | 0 | 0 | 0 | 0 | 14 |
| *Sphyrna mokarran* | 0 | 1 | 0 | 0 | 2 | 0 | 1 | 2 | 0 | 0 | 1 | 2 | 0 | 0 | 0 | 9 |
| *Stegostoma tigrinum* | 0 | 0 | 0 | 0 | 0 | 0 | 0 | 0 | 0 | 0 | 0 | 0 | 0 | 1 | 0 | 1 |
| *Stethojulis interrupta* | 2 | 0 | 0 | 0 | 0 | 0 | 0 | 0 | 0 | 0 | 0 | 0 | 0 | 0 | 0 | 2 |
| *Suezichthys sp* | 1 | 0 | 0 | 0 | 0 | 0 | 0 | 0 | 0 | 0 | 0 | 0 | 0 | 0 | 0 | 1 |
| *Sufflamen fraenatum* | 4 | 0 | 0 | 0 | 0 | 0 | 0 | 0 | 0 | 0 | 0 | 0 | 1 | 0 | 0 | 5 |
| *Symphorus nematophorus* | 2 | 0 | 0 | 0 | 1 | 0 | 0 | 0 | 0 | 0 | 0 | 0 | 1 | 0 | 0 | 4 |
| *Taeniurops meyeni* | 0 | 0 | 0 | 0 | 1 | 2 | 0 | 0 | 0 | 0 | 0 | 1 | 0 | 1 | 0 | 5 |
| *Terapon jarbua/ theraps* | 0 | 0 | 4 | 0 | 0 | 0 | 0 | 2 | 0 | 0 | 0 | 1 | 0 | 0 | 0 | 7 |
| *Torquigener* sp | 0 | 0 | 0 | 0 | 1 | 0 | 0 | 0 | 0 | 0 | 0 | 0 | 0 | 0 | 5 | 6 |
| *Trachinocephalus trachinus* | 0 | 0 | 0 | 0 | 0 | 0 | 0 | 0 | 0 | 0 | 0 | 0 | 1 | 0 | 0 | 1 |
| *Upeneus moluccensis* | 4 | 4 | 0 | 0 | 0 | 0 | 0 | 4 | 0 | 0 | 0 | 1 | 0 | 0 | 0 | 13 |
| *Variola* spp | 0 | 0 | 0 | 0 | 0 | 0 | 0 | 0 | 0 | 0 | 0 | 0 | 1 | 0 | 0 | 1 |
| *Wattsia mossambica* | 0 | 0 | 0 | 0 | 1 | 0 | 0 | 0 | 0 | 0 | 0 | 0 | 4 | 7 | 7 | 19 |
| *Xanthichthys lineopunctatus* | 3 | 0 | 0 | 0 | 0 | 0 | 0 | 0 | 0 | 0 | 0 | 0 | 0 | 0 | 0 | 3 |
| Grand Total | 288 | 87 | 85 | 46 | 256 | 167 | 175 | 249 | 99 | 252 | 249 | 130 | 317 | 279 | 195 | 2874 |
